# Examining the effect of lipid nanoparticle elasticity on endocytosis and mRNA delivery to cancer cells

**DOI:** 10.1007/s44258-026-00082-w

**Published:** 2026-05-01

**Authors:** Cecilia F. Shuler, Hannah C. Safford, Ajay S. Thatte, Melgious Ang, Michael J. Mitchell

**Affiliations:** 1 Department of Biophysics, University of Pennsylvania, Philadelphia, PA 19104 USA; 2 Department of Bioengineering, University of Pennsylvania, Philadelphia, PA 19104 USA; 3 Agency for Science, Technology and Research (A*STAR), Bioprocessing Technology Institute (BTI), Republic of Singapore, 138669 Singapore; 4 Perelman School of Medicine, Penn Institute for RNA Innovation, University of Pennsylvania, Philadelphia, PA 19104 USA; 5 Abramson Cancer Center, Perelman School of Medicine, University of Pennsylvania, Philadelphia, PA 19104 USA; 6 Institute for Immunology, Perelman School of Medicine, University of Pennsylvania, Philadelphia, PA 19104 USA; 7 Perelman School of Medicine, Cardiovascular Institute, University of Pennsylvania, Philadelphia, PA 19104 USA; 8 Institute for Regenerative Medicine, Perelman School of Medicine, University of Pennsylvania, Philadelphia, PA 19104 USA

**Keywords:** Lipid nanoparticles, Messenger RNA, Nanoparticle elasticity, Cancer

## Abstract

Lipid nanoparticles (LNPs) have emerged as modular and potent vehicles for nucleic acid delivery, offering a potentially promising new strategy for cancer therapy. This study investigates how LNP elasticity, modulated through variations in sterol lipid structure and sterol lipid molar ratio, affects LNP endocytosis and mRNA transfection in liver (HepG2), ovarian (OVCAR8), and lung (H1299) cancer cell lines. All LNP formulations were characterized for size, polydispersity index, and mRNA encapsulation efficiency and subsequently assessed for mRNA transfection in HepG2, OVCAR8, and H1299 cancer cells. In vitro screening showed that LNP elasticity influences mRNA transfection across all three cell types. Specifically, LNPs of intermediate stiffness enhanced mRNA transfection in HepG2 and OVCAR8 cells whereas LNPs with low stiffness improved mRNA transfection in H1299 cells. Mechanistic endocytosis studies showed that clathrin-mediated and lipid raft-mediated endocytosis pathways contribute to LNP uptake in all three cell lines. These findings highlight the promise of tuning LNP elasticity to enhance mRNA delivery to cancer cells and potentially improve the therapeutic efficacy of LNPs for cancer therapy applications.

**Graphical Abstract:**

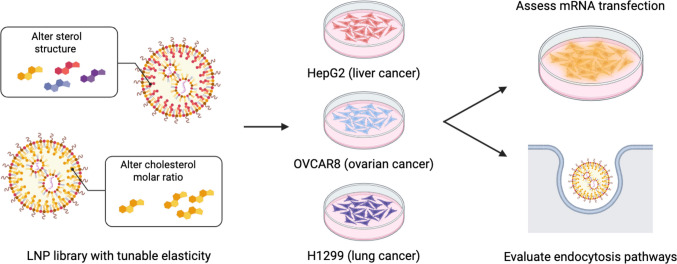

**Supplementary Information:**

The online version contains supplementary material available at 10.1007/s44258-026-00082-w.

## Highlights

- This study evaluates the role of lipid nanoparticle (LNP) elasticity on messenger RNA (mRNA) delivery to cancer cells.
- LNPs with low to intermediate stiffness enhance mRNA delivery in liver, ovarian and lung cancer cells.
- Clathrin-mediated and lipid raft-mediated endocytosis pathways contribute to LNP uptake in all three cancer cell lines.

**Supplementary Information:**

The online version contains supplementary material available at 10.1007/s44258-026-00082-w.

## Introduction

Cancer remains a significant global health burden with millions of new cases diagnosed each year [1]. Current treatment strategies primarily involve surgical resection in combination with chemotherapy or radiation therapy, but these therapies often incur systemic toxicity and off-target effects as they work by damaging rapidly dividing cells with minimal specificity [2, 3]. These limitations underscore the urgent need for more precise and less harmful therapeutic approaches.

Lipid nanoparticles (LNPs) have emerged as a transformative platform for RNA delivery across a range of therapeutic applications, including vaccines, genome editing and protein replacement therapies [4, 5]. Their ability to encapsulate and enable potent intracellular delivery of diverse RNA cargos has also made them a promising tool for cancer therapy [6, 7]. Specifically, LNP-mediated delivery of several RNA modalities has been investigated, including small interfering RNA (siRNA) and microRNA (miRNA) to silence oncogenic drivers, self-amplifying RNA (saRNA) to up-regulate tumor-suppressor pathways, and messenger RNA (mRNA) encoding tumor antigens for cancer vaccines or cytokines for immunotherapy [8–14]. Together, these strategies highlight the broad spectrum of RNA payloads under investigation for cancer therapies.

**Fig. 1:**
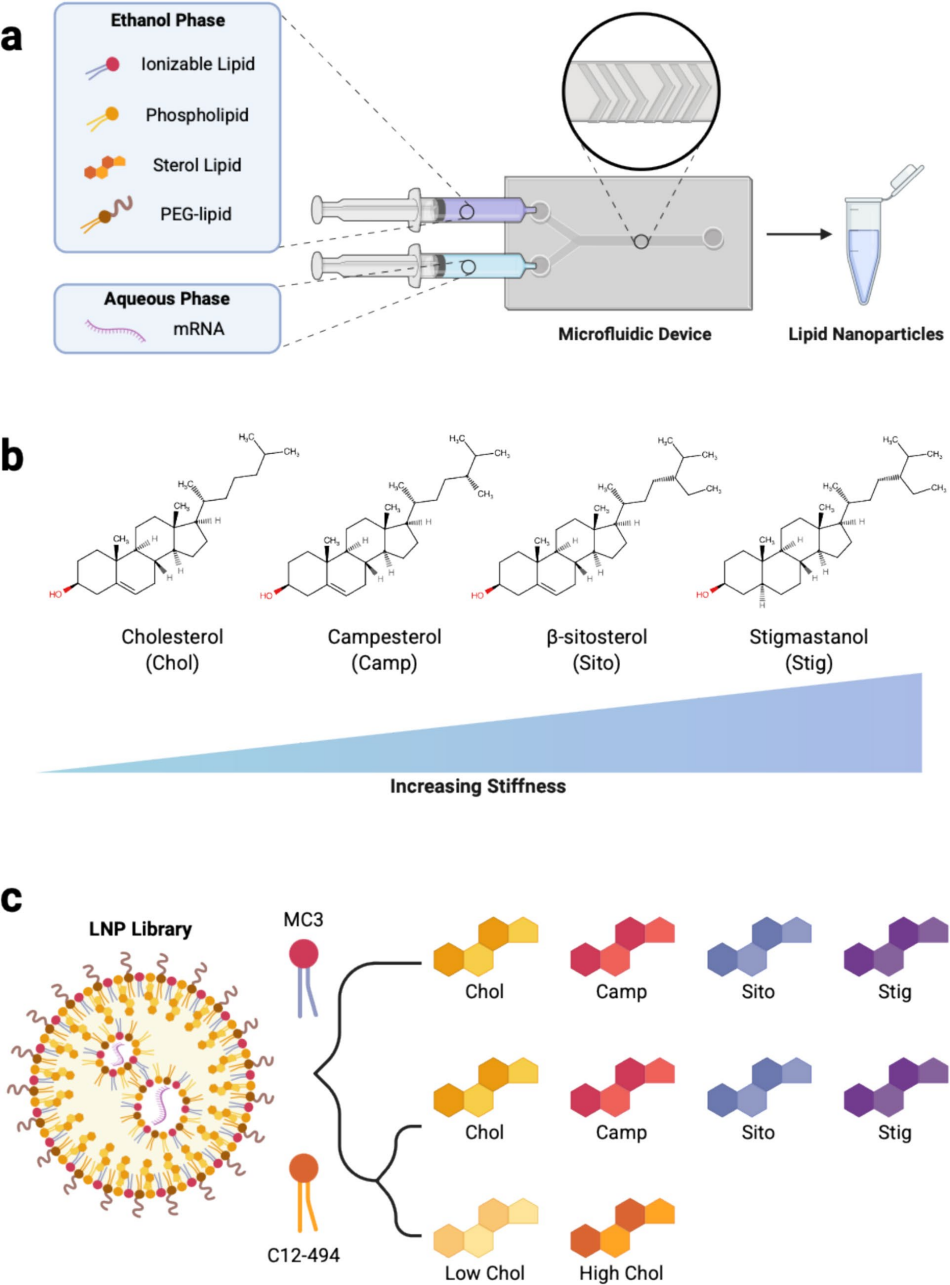
**a** Schematic of lipid nanoparticle (LNP) synthesis where an ethanol phase containing an ionizable lipid, phospholipid, sterol lipid and PEG-lipid is chaotically mixed with an aqueous phase containing mRNA in a microfluidic device. **b** Structures of cholesterol analogs used in this study (cholesterol, campesterol, β-sitosterol, and stigmastanol) and their intended effect on LNP stiffness. **c** Schematic of the LNP library design where LNPs are formulated with the ionizable lipid MC3 and one of four sterol lipids, the ionizable lipid C12-494 and one of four sterol lipids, or the ionizable lipid C12-494 with varying molar ratios of cholesterol

LNPs are typically composed of four excipients: an ionizable lipid, phospholipid, a sterol lipid, and lipid-anchored poly(ethylene glycol) (PEG). Each component plays an important role in promoting efficient mRNA encapsulation, enhancing LNP stability and facilitating endosomal escape to enable potent cytosolic delivery of the nucleic acid cargo **(**Fig. 1a) [15, 16]. Moreover, the modular nature of LNPs enables optimization of their chemical composition by tuning the excipient molar ratio or altering the excipients used. Such adjustments can facilitate targeted delivery to diseased tissues while minimizing systemic exposure, benefits that are particularly advantageous in cancer therapy, where improved specificity could reduce the extensive side effects associated with conventional cancer therapies [6, 17]. Previous studies have demonstrated that modifying the structure of the ionizable lipid, varying excipient molar ratios, or incorporating targeting moieties such as antibodies onto the LNP surface can alter LNP biodistribution and direct uptake to tumor sites [6, 18].

Beyond modulating the chemical composition of LNPs, studies have shown that tuning nanoparticle physical properties, such as nanoparticle elasticity, can influence nanoparticle-cell interactions. In the context of cancer, several studies have demonstrated that nanoparticle elasticity affects cellular uptake and tumor accumulation. For example, studies using core–shell polymeric nanoparticles found that softer nanoparticles were internalized more rapidly by prostate and colorectal cancer cells compared to their stiffer counterparts [19]. Similarly, nanolipogels with tunable elasticity showed enhanced cellular uptake and preferential accumulation in breast cancer cells when formulated to have softer mechanical properties [20]. Wu et. al. further reported that liposomes with moderate stiffness exhibited improved tumor penetration and diffusivity [21].

Many of these studies have examined elasticity in the context of other lipid- and polymer-based nanoparticles, but this concept has not been broadly explored for LNPs. To date, the role of LNP elasticity has not been extensively characterized, though it has been hypothesized to influence LNP interactions with cellular barriers and subsequent mRNA transfection [22, 23]. One study using atomic force microscopy reported that LNPs encapsulating mRNA were less stiff than those encapsulating plasmid DNA, although no Young’s modulus values were reported [24]. Additionally, the direct impact of LNP elasticity on LNP uptake and mRNA delivery in cancer cells remains unexplored.

Recent work by our group found that LNP elasticity can be tuned by incorporating different cholesterol analogs or by altering the molar ratio of cholesterol in the LNP formulation [25]. Specifically, we previously showed that LNPs can be engineered to exhibit Young’s moduli between 50–400 kPa, as measured by atomic force microscopy, by altering the ionizable lipid, substituting cholesterol with cholesterol analogs, or varying the cholesterol molar ratio [25]. In our previous work, we found that LNPs formulated with the ionizable lipid MC3 were generally softer than those formulated with the C12-494 ionizable lipid, and among all formulations, LNPs containing cholesterol exhibited the lowest Young’s modulus [25]. In contrast, incorporating cholesterol analogs, specifically campesterol, β-sitosterol, and stigmastanol increased the Young’s modulus (Fig. 1b) [25]. LNPs formulated with campesterol showed a moderate increase in Young’s modulus, while those formulated with β-sitosterol and stigmastanol exhibited approximately two-fold and four-fold higher moduli, respectively, compared to cholesterol-containing LNPs [25]. These changes in elasticity were hypothesized to arise from alkyl substitutions along the tail of each cholesterol analog, which are thought to disrupt lipid bilayer ordering and promote the formation of a more crystalline, faceted LNP structure [22, 26–28]. Additionally, decreasing the amount of cholesterol in our formulation produced softer LNPs, whereas increasing the amount of cholesterol generated stiffer LNPs, consistent with the known role of cholesterol in modulating lipid membrane fluidity [17, 22].

Here, we aim to explore the effect of LNP elasticity, through modulation of the sterol structure or cholesterol molar ratio, on mRNA transfection in liver (HepG2), ovarian (OVCAR8), and lung (H1299) cancer cells to assess if LNP elasticity can be tuned to improve mRNA LNP delivery to different cancer cell types. To this end, we formulated three LNP libraries: MC3 LNPs formulated with cholesterol analogs, C12-494 LNPs formulated with cholesterol analogs, and C12-494 LNPs formulated with varying cholesterol molar ratios (Fig. 1c). Each LNP library was screened in vitro across all three cancer cell lines. LNP elasticity was found to influence mRNA transfection efficiency, where LNPs of an intermediate stiffness enhanced mRNA delivery in HepG2 and OVCAR8 cells while LNPs with low stiffness mediated greater mRNA delivery in H1299 cells. Endocytosis pathway analysis further showed that clathrin-mediated and lipid raft-mediated mechanisms contribute to LNP uptake across all three cell types. Collectively, these findings offer insight into how both physical and compositional properties of LNPs influence their delivery efficiency in different cancer cell types.

## Results and discussion

### Formulation and characterization of LNPs

Thus far, limited work has explored the role of LNP elasticity on mRNA delivery to cancer cells. As such, we utilized a previously characterized LNP library that exhibited tunable elastic properties to evaluate whether differences in LNP elasticity influence mRNA transfection in liver, ovarian and lung cancer cells. To formulate LNPs, a lipid phase containing an ionizable lipid, phospholipid, sterol lipid and PEG-lipid were combined in ethanol at specific molar ratios and mixed in a microfluidic device with luciferase mRNA (Table S1). Two ionizable lipids were investigated: MC3, an FDA-approved ionizable lipid known for its potent RNA delivery to liver cells, and C12-494, which has demonstrated potent mRNA transfection across diverse cell types, including immune cells, placental cells, and brain endothelial cells [4, 29–33]. Each LNP was formulated with one of four sterol lipids, cholesterol (Chol), campesterol (Camp), β-sitosterol (Sito) or stigmastanol (Stig), or with varying molar ratios of cholesterol (Low Chol and High Chol). We elected to vary only the cholesterol molar ratio within the C12-494 LNP formulation, given the  expected potent transfection efficiency of this ionizable lipid across all three cell lines.

After formulation, all LNPs were characterized for size, polydispersity index (PDI), encapsulation efficiency, and zeta potential, as altering the sterol structure or cholesterol molar ratio can also affect other LNP physicochemical properties beyond elasticity, which may influence LNP performance (Table S2). The Z-average sizes ranged from 70.6–87.7 nm, with all LNPs exhibiting PDIs below 0.31, indicating uniform particle distribution. While most LNPs showed comparable sizes, MC3 Sito and Stig LNPs exhibited a modest ~ 10 nm size increase compared to the MC3 Chol LNP formulation. Similarly, the C12-494 Sito LNPs and C12-494 High Chol LNPs displayed slightly larger sizes over the C12-494 Chol formulation. All LNPs exhibited mRNA encapsulation efficiencies above 90%, suggesting that neither cholesterol analog incorporation nor variation in cholesterol molar ratio adversely affected mRNA loading. Lastly, all LNP formulations exhibited slightly negative zeta potentials. The C12-494 LNP library exhibited near-neutral zeta potentials, ranging from −2.70 to −8.24 mV, whereas the MC3 LNP library displayed more negative zeta potentials, between −13.56 and −21.09 mV. Overall, incorporation of different sterol analogs or variations in cholesterol molar ratio did not produce notable changes in LNP size, PDI, mRNA encapsulation efficiency, or zeta potential.

### Evaluation of LNP elasticity on in vitro mRNA transfection and LNP endocytosis in HepG2 cells

Nanoparticle elasticity has been shown to influence nanoparticle uptake in cancer cells, with softer nanoparticles demonstrating preferential tumor accumulation, enhanced cellular uptake and prolonged circulation times [20, 34]. As such, we sought to explore whether similar trends apply to LNPs and how LNP elasticity affects mRNA transfection in three cancer cell lines of epithelial origin: HepG2 cells, a model of hepatocellular carcinoma, OVCAR8 cells, a model of ovarian cancer, and H1299, a non-small cell lung carcinoma cell line. These cell lines were chosen because they are well-established cancer models and have been previously explored in LNP delivery studies [35–40].

**Fig. 2:**
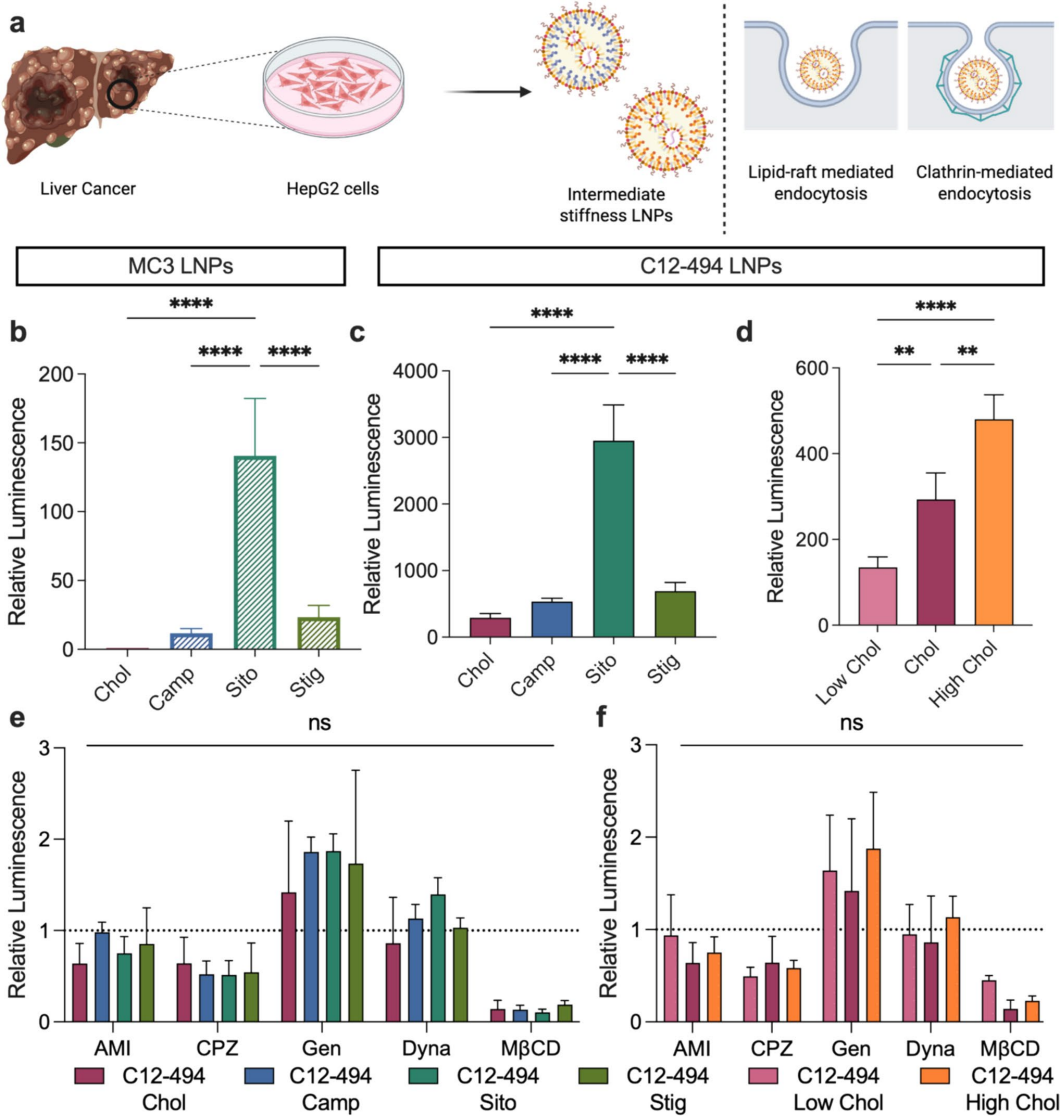
Investigating the role of LNP elasticity on mRNA transfection and LNP endocytosis in HepG2 liver cancer cells. **a** Overall schematic highlighting that LNPs of an intermediate stiffness potently transfect HepG2 cells via lipid-raft mediated and clathrin-mediated endocytosis. Luciferase expression in HepG2 cells 24 h after treatment with **b** MC3 LNPs incorporating each cholesterol analog, **c** C12-494 LNPs incorporating each cholesterol analog or **d** C12-494 LNPs with decreased (Low) or increased (High) molar amounts of cholesterol at a dose of 10 ng of mRNA per 10,000 cells. Relative luminescence was quantified by normalizing to cells treated with MC3 Chol LNPs. Results are reported as mean ± standard deviation from 4 biological replicates. A nested one-way ANOVA with post hoc Student’s t tests using the Holm- Šídák correction for multiple comparisons was used to compare relative luminescence across treatment groups to the MC3 or C12-494 Chol LNP, **p ≤ 0.01, ****p ≤ 0.0001, ns = not significant. Relative luciferase expression in HepG2 cells 24 h after treatment with **e** C12-494 LNPs incorporating each cholesterol analog or **f** C12-494 Low and High cholesterol LNPs at a dose of 10 ng of mRNA per 10,000 cells in the presence of different endocytosis inhibitors (Amiloride (AMI) is an inhibitor of macropinocytosis; chlorpromazine (CPZ) is an inhibitor of clathrin-mediated endocytosis; genistein (GEN) is an inhibitor of caveolae-mediated endocytosis; dynasore (DYNA) is an inhibitor of dynamin-dependent endocytosis; methyl-β-cyclodextrin (MβCD) is an inhibitor of lipid-raft mediated endocytosis). Relative luminescence signal was quantified by normalizing to cells treated with LNPs in the absence of endocytosis inhibitors. Results are reported as mean ± standard deviation from 3 biological replicates. A two-way ANOVA with post hoc Student’s t tests using the Holm-Šídák correction for multiple comparisons was used to compare luciferase expression across treatment groups and inhibitors to the C12-494 Chol LNP

For in vitro screening, LNPs were formulated with luciferase mRNA as a reporter mRNA cargo. To assess mRNA delivery in liver cancer cells, HepG2 cells were treated with the three LNP libraries at a dose of 10 ng of luciferase mRNA per 10,000 cells. Twenty-four h after LNP treatment, luciferase expression and cell viability were measured and compared to the MC3 or C12-494 cholesterol LNP formulations **(**Fig. 2a**)**. Luminescence signal following LNP treatment is reported as a relative luminescence value normalized to the control LNP formulation, MC3 Chol. While this approach allows for comparison across treatment groups, the measured luminescence signal reflects average luciferase expression across the entire cell population rather than individual transfection events, which can obscure heterogeneity in transfection within the cell population.

Within the MC3 LNP library, the MC3 Sito LNP mediated significantly higher luciferase expression than the MC3 Chol, Camp and Stig LNPs (Fig. 2b). This trend was mirrored in the C12-494 LNP library, where C12-494 Sito LNPs also demonstrated significantly enhanced mRNA transfection efficiency relative to the other C12-494 cholesterol analog formulations, highlighting the consistent benefit of β-sitosterol across different ionizable lipids (Fig. 2c). In addition, the C12-494 High Chol LNPs mediated significantly greater transfection than both the C12-494 Low Chol and Chol formulations and the C12-494 Chol LNP outperformed the C12-494 Low Chol formulation (Fig. 2d). Finally, negligible cytotoxicity was observed with any of the LNP formulations in the HepG2 cells (Fig. S1). To further assess potential toxicity of the LNP library, RAW-Blue reporter cells were treated with each LNP and NF-κB activity was measured. None of the LNPs induced significantly higher NF-κB activity compared to untreated cells; however, cells treated with C12-494 LNPs showed significantly higher NF-κB activity compared to cells treated with MC3 LNPs, suggesting that C12-494 LNPs may illicit a more pronounced inflammatory response (Fig. S2).

Overall, these results suggest that mRNA delivery to HepG2 cells improves when LNP stiffness is increased. In both the MC3 and C12-494 cholesterol analog libraries, mRNA transfection was enhanced when LNP stiffness was increased through cholesterol analog incorporation; however, LNPs of an intermediate stiffness, those formulated with β-sitosterol, exhibited the greatest improvement in mRNA transfection compared to the softer cholesterol formulations. A similar trend was observed in the C12-494 library with varied cholesterol molar ratios, where LNPs containing a higher cholesterol content and consequently higher Young’s modulus mediated more potent mRNA transfection. These findings align with previous hypotheses suggesting that while increased LNP stiffness can enhance mRNA delivery, excessively stiff LNPs, such as those formulated with stigmastanol in both the MC3 and C12-494 libraries, may exhibit reduced cellular uptake or impeded mRNA release due to enhanced stability in the biological environment [22, 25].

Next, to investigate factors contributing to the enhanced mRNA transfection observed with the C12-494 Sito and High Chol LNPs compared to the Chol LNP formulation, we examined LNP colocalization within the endosomal compartments of HepG2 cells using confocal microscopy (Fig. S3a). At 2 h post-treatment with LNPs, we did not observe differences in endosomal escape among the LNPs, which was further supported by comparable Pearson correlation coefficients (Fig. S3b-c). While differences in endosomal scape were not detected at this time point, it is possible that key endosomal escape events occur later than 2 h in HepG2 cells. Additionally, although overall endosomal escape rates appeared low, the strong colocalization signal and high Pearson correlation coefficients for the C12-494 Sito and High Chol LNPs suggest that these LNPs may remain in endosomes longer, potentially trafficking them to more favorable compartments such as the late endosome, which has been previously linked to mRNA release, including for LNPs containing sterol analogs [26, 41, 42]. Finally, the strong LNP signal observed in the C12-494 Sito and High Chol microscopy images suggests robust cellular uptake, which may also contribute to the enhanced mRNA transfection in vitro.

To further probe the uptake mechanisms contributing to LNP-mediated mRNA delivery, HepG2 cells were pre-treated with various endocytosis inhibitors prior to luciferase mRNA LNP transfection. Previous studies have shown that nanoparticle elasticity influences uptake pathways, where stiffer nanoparticles are often internalized via clathrin-mediated endocytosis while softer nanoparticles are endocytosed through non-specific fusion mechanisms [20, 21, 43]. To explore the role of LNP elasticity on endocytosis, we tested five small molecule inhibitors targeting different endocytosis pathways. Specifically, we tested amiloride, an inhibitor of macropinocytosis, chlorpromazine, an inhibitor of clathrin-mediated endocytosis, genistein, an inhibitor of caveolae-mediated endocytosis, dynasore, an inhibitor of dynamin-mediated endocytosis, and methyl-β-cyclodextrin, an inhibitor of lipid-raft mediated endocytosis (Fig. S4).

To investigate these endocytosis pathways, LNPs encapsulating luciferase mRNA were used to treat HepG2 cells that had been pre-treated with each inhibitor. Given that the C12-494 LNP library mediated higher mRNA transfection in HepG2 cells compared to the MC3 library, only the C12-494 LNPs were included in this study. While no significant differences were observed between the different cholesterol analog formulations for each inhibitor, all cholesterol analog LNPs showed a substantial reduction in mRNA transfection following methyl-β-cyclodextrin treatment, indicating that lipid-raft mediated endocytosis may be the primary uptake pathway for these formulations in liver cancer cells (Fig. 2e, Fig. S5). This pattern held true for cells treated with the LNP library with varied cholesterol content. Both the C12-494 Low Chol and High Chol LNPs saw substantial reduction in luminescence signal when the cells were pre-treated with methyl-β-cyclodextrin, highlighting lipid-raft mediated endocytosis as a primary mechanism for these LNPs to achieve uptake in HepG2 cells (Fig. 2f, Fig. S5). Interestingly, the C12-494 Low Chol LNP only exhibited a 50% reduction in luminescence signal in the presence of methyl-β-cyclodextrin, compared to roughly 20% for the C12-494 Chol and High Chol LNPs, suggesting that this formulation may not use lipid-raft mediated pathways to the same degree as other LNP formulations. In addition, nearly all LNP treatment groups saw significant luminescence inhibition in the presence of chlorpromazine, suggesting these LNPs may also rely on clathrin-mediated pathways for endocytosis (Fig. S5).

While these inhibitors can help indicate which pathways contribute to LNP uptake, their effects on endocytic processes are often non-specific [44]. For example, amiloride modulates macropinocytosis through sodium channel and pH regulation, but it can also affect fast endophilin-mediated endocytosis [44, 45]. Chlorpromazine disrupts clathrin-mediated endocytosis but also affects dynamin-dependent processes [46]. Genistein, commonly used to block caveolae-mediated uptake, broadly inhibits tyrosine-specific protein kinases and can also impact clathrin-dependent internalization [44, 47, 48]. Dynasore has been reported to alter cellular signaling, including mTORC1 activation, and reduce membrane cholesterol, thereby disrupting lipid rafts [48]. Finally, methyl-β-cyclodextrin inhibits lipid-raft mediated uptake by depleting membrane cholesterol, but this also affects cell membrane structure, fluidity and signaling organization [44, 49]. Although these broader cellular effects require that results be interpreted within the context of each inhibitor’s mechanism, the overall inhibition patterns can still provide useful insights into the pathways most likely contributing the LNP uptake in specific cell lines.

### Evaluation of LNP elasticity on in vitro mRNA transfection and LNP endocytosis in OVCAR8 cells

**Fig. 3:**
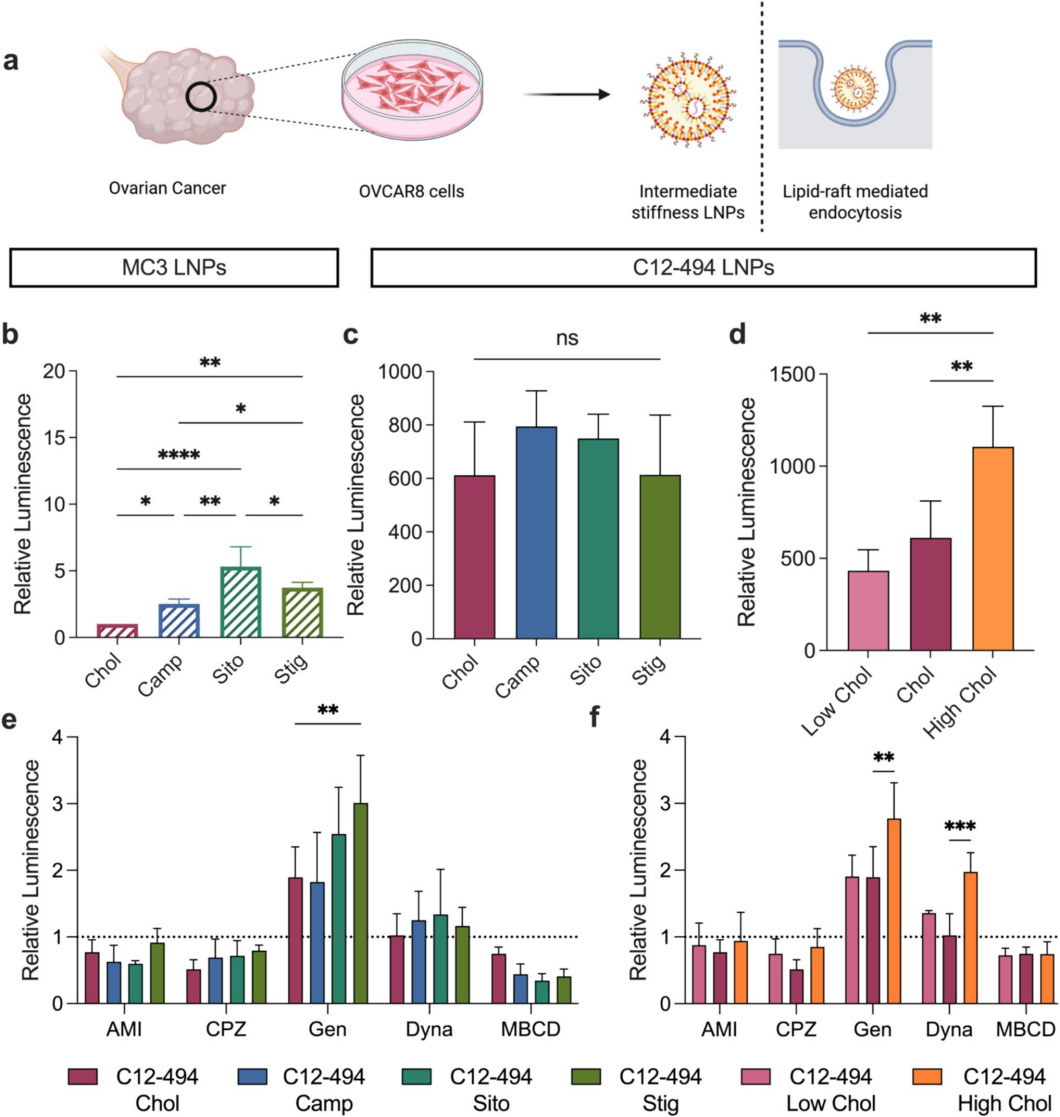
Investigating the role of LNP elasticity on mRNA transfection and LNP endocytosis in OVCAR8 ovarian cancer cells. **a** Overall schematic highlighting that LNPs of an intermediate stiffness potently transfect OVCAR8 cells via lipid-raft mediated endocytosis. Luciferase expression in OVCAR8 cells 24 h after treatment with **b** MC3 LNPs incorporating each cholesterol analog, **c** C12-494 LNPs incorporating each cholesterol analog or **d** C12-494 LNPs with decreased (Low) or increased (High) molar amounts of cholesterol at a dose of 10 ng of mRNA per 20,000 cells. Relative luminescence was quantified by normalizing to cells treated with MC3 Chol LNPs. Results are reported as mean ± standard deviation from *n* = 4 biological replicates. A nested one-way ANOVA with post hoc Student’s t tests using the Holm-Šídák correction for multiple comparisons was used to compare relative luminescence across treatment groups to the MC3 or C12-494 Chol LNP, *p ≤ 0.05, **p ≤ 0.01, ***p ≤ 0.001, ****p ≤ 0.0001, ns = not significant. Relative luciferase expression in OVCAR8 cells 24 h after treatment with **e** C12-494 LNPs incorporating each cholesterol analog or **f** C12-494 Low and High cholesterol LNPs at a dose of 10 ng of mRNA per 20,000 cells in the presence of different endocytosis inhibitors (Amiloride (AMI), chlorpromazine (CPZ), genistein (GEN), dynasore (DYNA), methyl-β-cyclodextrin (MβCD)). Relative luminescence signal was quantified by normalizing to cells treated with LNPs in the absence of endocytosis inhibitors. Results are reported as mean ± standard deviation from 3 biological replicates. A two-way ANOVA with post hoc Student’s t tests using the Holm-Šídák correction for multiple comparisons was used to compare luciferase expression across treatment groups and inhibitors to the C12-494 Chol LNP, **p ≤ 0.01, ***p ≤ 0.001

Since the C12-494 ionizable lipid has been shown to mediate mRNA transfection in female reproductive organs, including the ovaries, uterus and placenta, we sought to explore the effect of LNP elasticity on mRNA transfection in ovarian cancer cells using both the C12-494 and MC3 LNP libraries [30]. To assess mRNA transfection in a model of ovarian cancer, OVCAR8 cells were treated with the three LNP libraries at a dose of 25 ng of luciferase mRNA per 20,000 cells. Twenty-four h after LNP treatment, luciferase expression and cell viability were measured for all treatment groups and compared to either the MC3 or C12-494 cholesterol LNP formulations (Fig. 3a).

Within the MC3 LNP library, incorporating cholesterol analogs and increasing LNP stiffness improved mRNA delivery to OVCAR8 cells compared to the cholesterol formulation. Notably, the MC3 Sito formulation mediated significantly higher luciferase expression compared to all other formulations (Fig. 3b). In contrast, the C12-494 LNP library exhibited smaller differences in mRNA transfection between each cholesterol analog (Fig. 3c). Within this library, the C12-494 Camp and Sito formulations exhibited modest improvements in luciferase signal compared to C12-494 Chol and Stig LNPs, again suggesting that LNPs with intermediate stiffness may improve mRNA delivery to ovarian cancer cells. Importantly, no cytotoxicity was observed for any of the tested LNP formulations (Fig. S6).

Similar to the results in HepG2 cells, the stiffer, C12-494 High Chol LNP mediated significantly improved mRNA delivery in OVCAR8 cells compared to the low stiffness, C12-494 Low Chol and Chol formulations (Fig. 3d). Additionally, the relative luminescence signal for all C12-494 LNPs was 50- to 100- fold higher than that of the MC3 LNPs, highlighting the potency of this ionizable lipid in transfecting cells from female reproductive tissues. Collectively, these results suggest that increasing LNP stiffness improves mRNA transfection in OVCAR8 cells and that the C12-494 High Chol LNP mediates potent mRNA transfection in ovarian cancer cells.

We again examined if differences in mRNA transfection between formulations could be a result of differences in endosomal escape. Using confocal microscopy, we assessed LNP colocalization within the endosomal compartments of OVCAR8 cells for the two strongest performing LNP formulations, C12-494 Camp and High Chol, compared to the C12-494 Chol LNP (Fig. S7a). At 2 h post-treatment with LNPs, no differences in endosomal escape or Pearson correlation coefficients were observed between the formulations (Fig. S7b, c). The overall low rates of endosomal escape for all three formulations suggest that endosomal escape may occur at later time points in OVCAR8 cells, potentially showing differences beyond 2 h. Additionally, the lead LNP formulations, particularly the C12-494 High Chol LNP, exhibited stronger LNP signal compared to the cells treated with C12-494 Chol LNPs, indicating enhanced cellular uptake and potentially contributing to more efficient downstream mRNA transfection.

To investigate uptake pathways involved in mRNA transfection, OVCAR8 cells were pre-treated with the same panel of endocytosis inhibitors previously described prior to treatment with the C12-494 LNP libraries encapsulating luciferase mRNA. Due to their low transfection efficiency, MC3 LNPs were again excluded from this study. Across both libraries, endocytosis inhibition was not observed when pre-treated with genistein and dynasore, inhibitors of caveolar and dynamin-dependent endocytosis pathways, respectively, suggesting these pathways do not significantly contribute to LNP uptake in ovarian cancer cells (Fig. 3e, f). Interestingly, luminescence signal increased significantly when OVCAR8 cells were pre-treated with genistein and dosed with the stiffer Stig and High Chol LNPs, indicating that blocking caveolae-mediated endocytosis may enhance LNP uptake and mRNA delivery in ovarian cancer cells. No significant differences in luminescence signal between LNP formulations were observed with amiloride or chlorpromazine pre-treatment. For nearly all LNP formulations, pre-treatment of OVCAR8 cells with methyl-β-cyclodextrin led to a significant reduction in luminescence signal (Fig. S8). Although not statistically significant, C12-494 LNPs formulated with cholesterol analogs showed a trend toward greater inhibition in the presence of methyl-β-cyclodextrin compared to the softer C12-494 Chol LNP, suggesting these LNP formulations may rely more on lipid-raft mediated endocytosis for uptake in ovarian cancer cells (Fig. 3e).

### Evaluation of LNP elasticity on in vitro mRNA transfection and LNP endocytosis in H1299 cells

**Fig. 4:**
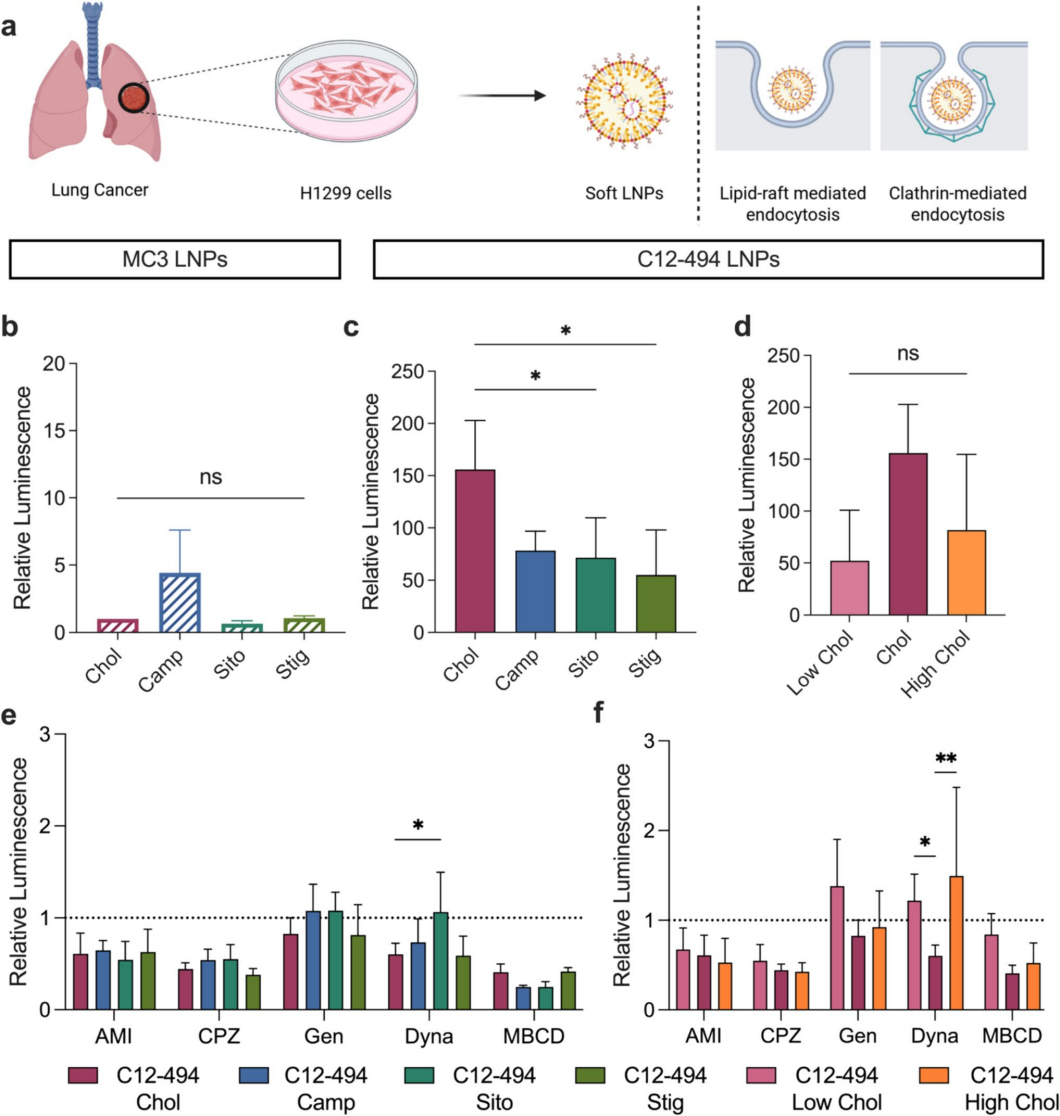
Investigating the role of LNP elasticity on mRNA transfection and LNP endocytosis in H1299 lung cancer cells. **a** Overall schematic highlighting that softer LNPs potently transfect H1299 cells via lipid-raft and clathrin-mediated endocytosis. Luciferase expression in H1299 cells 24 h after treatment with **b** MC3 LNPs incorporating each cholesterol analog, **c** C12-494 LNPs incorporating each cholesterol analog or **d** C12-494 LNPs with decreased (Low) or increased (High) molar amounts of cholesterol at a dose of 20 ng of mRNA per 20,000 cells. Relative luminescence was quantified by normalizing to cells treated with MC3 Chol LNPs. Results are reported as mean ± standard deviation from 3 biological replicates. A nested one-way ANOVA with post hoc Student’s t tests using the Holm- Šídák correction for multiple comparisons was used to compare relative luminescence across treatment groups to the MC3 or C12-494 Chol LNP, *p ≤ 0.05, ns = not significant. Relative luciferase expression in H1299 cells 24 h after treatment of **e** C12-494 LNPs incorporating each cholesterol analog or **f** C12-494 Low and High cholesterol LNPs at a dose of 10 ng of mRNA per 10,000 cells in the presence of different endocytosis inhibitors (Amiloride (AMI), chlorpromazine (CPZ), genistein (GEN), dynasore (DYNA), methyl-β-cyclodextrin (MβCD)). Relative luminescence signal was quantified by normalizing to cells treated with LNPs in the absence of endocytosis inhibitors. Results are reported as mean ± standard deviation from 3 biological replicates. A two-way ANOVA with post hoc Student’s t tests using the Holm-Šídák correction for multiple comparisons was used to compare luciferase expression across treatment groups and inhibitors to the C12-494 Chol LNP, *p ≤ 0.05, **p ≤ 0.01

mRNA LNPs have been explored for the treatment of lung cancer mouse models and as such, we sought to evaluate if altering LNP elasticity could influence LNP-mediated mRNA delivery in a lung cancer cell line [50, 51]. To assess mRNA delivery in a model of lung cancer, H1299 cells were treated with the three LNP libraries at a dose of 20 ng of luciferase mRNA per 20,000 cells. Twenty-four h after LNP treatment, luciferase expression and cell viability were measured for all treatment groups and compared to either the MC3 or C12-494 cholesterol LNP formulations (Fig. 4a).

Across both the MC3 and C12-494 cholesterol analog libraries, LNP formulations with low stiffness outperformed their stiffer counterparts (Fig. 4b, c). Specifically, the MC3 Camp LNP exhibited a four-fold improvement in mRNA delivery compared to the other MC3 formulations, while the C12-494 Chol LNP mediated significantly higher mRNA transfection compared to the C12-494 Sito and Stig LNPs. Altering the LNP elasticity by varying the cholesterol molar ratio in C12-494 LNPs did not significantly affect mRNA delivery in the H1299 cells (Fig. 4d). Consistent with findings in HepG2 and OVCAR8 results, all C12-494 LNPs achieved an order of magnitude greater mRNA expression than the MC3 LNPs, underscoring the potency of this ionizable lipid for cancer cell transfection. No cytotoxicity was observed for any of the LNPs tested (Fig. S9).

We next examined differences in LNP uptake and endosomal escape between the low stiffness C12-494 Chol LNPs and the stiffer C12-494 Stig and High Chol LNP formulations using confocal microscopy in H1299 cells (Fig. S10a). Although statistically significant differences in endosomal escape were not observed across formulations, the C12-494 Stig LNPs exhibited a threefold higher rate of endosomal escape compared to the C12-494 Chol LNP, despite showing poor mRNA transfection in H1299 cells (Fig. S10b). In addition, we observed strong LNP signal in the cells treated with C12-494 Chol LNPs, suggesting that while endosomal escape rates may be low, higher LNP accumulation may contribute to the enhanced mRNA transfection observed for this formulation. All three LNP formulations had Pearson correlation coefficients greater than 0.7, indicating strong co-localization with endosomal compartments and suggesting that additional endosomal escape events may occur at later time points (Fig. S10c).

To further understand LNP uptake mechanisms, H1299 cells were pre-treated with the same panel of endocytosis inhibitors previously described prior to treatment with the C12-494 LNP libraries encapsulating luciferase mRNA. MC3 LNPs were again excluded due to their lower transfection efficiency. Moderate inhibition of mRNA delivery was observed for all LNP treatment groups when H1299 cells were pre-treated with amiloride and chlorpromazine, inhibitors of macropinocytosis and clathrin-mediated endocytosis, respectively, indicating that all LNPs partially use these pathways independent of LNP elasticity (Fig. 4e, f, Fig. S11). Similarly, partial inhibition of endocytosis occurred for methyl-β-cyclodextrin pre-treatment, where greater inhibition was observed for stiffer LNPs compared to softer formulations, although there were no significant differences in inhibition between LNP groups. Minimal to no inhibition was observed with genistein pre-treatment, suggesting caveolae-mediated endocytosis is not a major uptake pathway in H1299 cells. Notably, differences in endocytosis inhibition emerged with dynasore pre-treatment, an inhibitor of dynamin-dependent endocytosis. The C12-494 Sito LNP showed no endocytosis inhibition in the presence of dynasore, which was significantly different from the inhibition observed for the C12-494 Chol LNP. Similarly, both the C12-494 Low Chol and High Chol LNPs saw no inhibition of mRNA translation in the presence of dynasore compared to the C12-494 Chol LNP. Overall, these results suggest that LNP endocytosis pathways vary when LNP elasticity is altered and that these pathways may differ for the same LNP formulation across different cell types.

## Conclusion

This work investigated the role of LNP elasticity, modulated by variations in sterol structure and cholesterol molar ratio, on mRNA transfection across three cancer cell models: hepatocellular carcinoma (HepG2), ovarian cancer (OVCAR8), and non-small cell lung cancer (H1299). Our findings demonstrate that LNP elasticity affects mRNA delivery efficiency, where LNPs of intermediate stiffness enhanced mRNA delivery in liver and ovarian cancer cells and LNPs with low stiffness improved mRNA transfection in lung cancer cells. Differences between the top-performing LNP formulations across each cell line may also be attributed to differences in the membrane composition and fluidity of each cancer cell, which in turn may affect LNP endocytosis [52, 53]. For example, H1299 cells have been reported to have relatively higher membrane cholesterol content, resulting in reduced membrane fluidity, which may explain their preference for softer LNPs [54]. Additionally, endocytosis inhibition studies showed that LNP uptake may occur primarily through clathrin-mediated and lipid-raft mediated endocytosis pathways across all three cell lines. These results suggest that tuning LNP elasticity is a promising strategy to optimize mRNA delivery to different cancer types, with the optimal balance likely residing in formulations of an intermediate stiffness. Notably, the top-performing LNPs, C12-494 Sito and High Chol, have measured Young’s moduli of 202 and 136 kPa, respectively, consistent with previous findings that nanoparticles with moduli in the kilopascal-to-megapascal range demonstrate enhanced nanoparticle uptake in cancer cells [20, 21, 25, 34].

In addition, future studies can further strengthen our understanding of how LNP elasticity influences nanoparticle uptake mechanisms and mRNA transfection across diverse cancer cell models. In this work, we utilized well-established cancer cell lines for nanoparticle studies; however, the findings here are limited by the fact that these models are all epithelial in origin. Future studies should evaluate LNPs with varied elasticity across a broader range of cancer cell types, including mesenchymal and hematological phenotypes. Additional studies should also investigate a broader panel of cholesterol analogs to determine whether specific structural features contribute to the improved performance of β-sitosterol LNPs in cancer cells and explore varying cholesterol molar ratios in other ionizable lipid LNP formulations, such as MC3. Future work should also include more comprehensive endocytosis studies, utilizing expanded inhibitor panels and siRNA-mediated knockdown of specific proteins involved in distinct endocytic pathways to further elucidate stiffness-dependent uptake mechanisms. Additionally, utilizing dye-labeled LNPs to assess inhibition of LNP uptake will help confirm that blocking these pathways affects both LNP internalization and downstream mRNA translation.

Finally, in vivo biodistribution and tumor model studies using therapeutic mRNA cargos will be critical to validate these LNP design principles and advance their translation into clinically relevant cancer therapies. While tumor microenvironments are considerably more complex than 2D in vitro cell culture systems, in vitro experiments provide valuable mechanistic insight into how LNP elasticity influences cellular uptake and mRNA transfection. Although in vitro and in vivo results do not always correlate, previous studies examining nanoparticle elasticity in the context of cancer have shown that candidates that perform well in vitro often translate to in vivo efficacy, suggesting that the findings here may translate in in vivo tumor studies [20, 21, 34]. Furthermore, the findings presented here establish trends between particle stiffness and transfection efficiency, enabling the elimination of formulations that exhibit poor performance or cytotoxicity. These findings can guide the selection of promising LNP candidates for in vivo evaluation, where parameters such as circulation time, tumor penetration, and therapeutic efficacy can be assessed within the context of the more physiologically relevant tumor microenvironment. Collectively, this work underscores the importance of nanoparticle elasticity as a LNP design parameter and provides a foundation for engineering LNPs with optimized mechanical properties to potentially improve the safety, efficacy, and specificity of mRNA-based cancer therapies.

## Materials and methods

### Ionizable lipid synthesis

The C12-494 ionizable lipid was synthesized as previously described [25, 30]. Briefly, the polyamine core 2-{2-[4-(2-{[2-(2 aminoethoxy)ethyl]amino}ethyl)piperazin-1-yl]ethoxy}ethan1-amine (Enamine, Kyiv, Ukraine) was reacted with an excess of the epoxide tail 1,2-epoxydodecane (MilliporeSigma, Burlington, MA) under gentle stirring for 48 h at 80ºC. Flash chromatography was used to purify the product and a Rotovapor R-300 rotary evaporator (Buchi, New Castle, DE) was utilized to dry the product before the lipid was resuspended in ethanol at a concentration of 40 mg/ml for LNP synthesis.

### LNP synthesis

To formulate LNPs, a lipid mixture comprising an ionizable lipid phospholipid, sterol lipid and PEG-lipid were combined in an ethanol phase at various molar ratios specified in Table S1. Specifically, the ionizable lipids C12-494 or MC3 (Cayman Chemical, Ann Arbor, MI)) were used along with the phospholipids 1,2-dioleoyl-sn-glycero-3-phosphoethanolamine (DOPE) or 1,2-distearoyl-sn-glycero-3-phosphocholine (DSPC) (Avanti Polar Lipids, Alabaster, AL)), sterol lipids cholesterol (Sigma Aldrich, St. Louis, MO), campesterol, β-sitosterol or stigmastanol (Cayman Chemical), and lipid-PEGs 1,2-dimyristoyl-sn-glycero-3-phosphoethanolamine-N-[methoxy(polyethylene glycol)−2000] (ammonium salt) (C14-PEG_2k_) or 1,2-dimyristoyl-rac- glycero-3-methoxypolyethylene glycol-2000 (DMG-PEG_2k_) (Avanti Polar Lipids). Pre-aliquoted mRNA with N^1^-methylpseudouridine modifications (Trilink Biotechnologies, San Diego, CA) was dissolved in 10 mM citric acid buffer (pH 3) to produce the aqueous phase. All LNPs were formulated at a weight ratio of 10:1 of ionizable lipid to mRNA. Syringe pumps (Harvard Apparatus, Holliston, MA) were used to combine the ethanol and aqueous phases via chaotic mixing in a microfluidic device at a 1:3 volumetric ratio. After synthesis, LNPs were dialyzed against 1X PBS for 2 h in dialysis cassettes with a 20 kDa molecular weight cut off (Thermo Fisher Scientific, Waltham, MA) and sterilized with 0.22 μm filters. The particles were then stored at 4 °C until further use.

### LNP characterization

LNP size and PDI were measured using dynamic light scattering (DLS) via a DynaPro® Plate Reader III (Wyatt Technology, Santa Barbara, CA). LNPs were diluted in 1X PBS for DLS measurements and for each sample, three measurements with at least five runs were recorded. Z-average size and PDI are reported as mean ± standard deviation of 3 replicates. Zeta potential measurements were conducted using a Zetasizer Nano (Malvern Instruments). LNPs were diluted in water at for zeta potential measurements, three measurements with at least 10 runs were recorded. Zeta potential measurements are reported as mean ± standard deviation of  3 replicates.

Encapsulation efficiency was quantified via a Quant-iT RiboGreen RNA assay (Thermo Fisher Scientific). Each LNP formulation was diluted 80-fold in either 1X tris–EDTA (TE) buffer or TE buffer with 0.1% Triton X-100 (Millipore Sigma). LNPs in TE or Triton X-100 and mRNA standards were plated in quadruplicate in black 96-well plates and the RiboGreen reagent was added to each well. After 5 min of incubation at room temperature, the fluorescence intensity was read on a plate reader at an excitation wavelength of 480 nm and an emission wavelength of 520 nm. Encapsulation efficiencies were calculated as [(RNA content in Triton X-100-RNA content in TE buffer)/RNA content in Triton X-100]*100. Encapsulation efficiencies are reported as mean ± standard deviation of  4 replicates.

### Cell culture

In vitro experiments were conducted using human HepG2 hepatocellular carcinoma (ATCC #HB-8065), human H1299 carcinoma (ATCC #CRL-5803) and human OVCAR8 cell lines. OVCAR8 cells were kindly provided by Dr. Ronny Drapkin (University of Pennsylvania). All cell lines tested negative for mycoplasma and the morphology of all cells was checked at every passage to ensure they were free from contamination. HepG2 cells were cultured in Dulbecco’s Modified Eagle Medium (DMEM, Gibco, Dublin, Ireland) supplemented with 10% FBS (Gibco) and 1% penicillin–streptomycin (Gibco). H1299 and OVCAR8 cells were cultured in Roswell Park Memorial Institute Medium (RPMI, Gibco) supplemented with 10% FBS (Gibco) and 1% penicillin–streptomycin (Gibco). All three cell lines were maintained at 37ºC and 5% CO_2_.

### In vitro LNP-mediated luciferase mRNA delivery to cancer cells

Luciferase assays were used to evaluate the efficiency of mRNA transfection by LNPs. To quantify LNP-mediated mRNA transfection, cells were plated in 100µL of media in 96‑well plates and left to adhere overnight. HepG2 and H1299 cells were plated at a concentration of 10,000 cells/well and OVCAR8 cells were plated at a concentration of 20,000 cells/well. HepG2 cells were treated with LNPs at a dose of 10 ng of luciferase mRNA, OVCAR8 cells were treated with LNPs at a dose of 25 ng of luciferase mRNA, and H1299 cells were treated with LNPs at a dose of 20 ng of luciferase mRNA. Twenty-four h after LNP treatment, the media was aspirated, and 50 µL of 0.1% Triton X‑100 was added to each well to lyse the cells. Plates were placed on a shaker for 2–3 min to ensure lysis. Subsequently, 100 µL of luciferin substrate (Promega, Madison, WI) was added to each well and incubated for 10 min at room temperature with gentle shaking. Luminescence was measured using a plate reader (Tecan, Morrisville, NC). Relative luminescence signal was calculated by first subtracting the background readings from untreated cells and then dividing by the average luminescence signal of cells treated with MC3 Chol LNPs. Relative luciferase expression is reported as the mean ± standard deviation of 3 or 4 biological replicates (averaged from 4 technical replicates). A nested one-way ANOVA with post hoc Student’s t tests using the Holm-Šídák correction for multiple comparisons were used to compare luciferase expression across treatment groups.

Cytotoxicity of LNPs was evaluated using the CellTiter‑Glo® Luminescent Cell Viability Assay. To quantify cytotoxicity of LNPs after 24 h of treatment, cells were plated in 100 ul of media in 96‑well plates at the seeding densities described above and left to adhere overnight. Cells were treated at the same luciferase mRNA LNP dose described above and 24 h after dosing, 100 µL of CellTiter‑Glo® (Promega) was added directly to each well and incubated for 10 min at room temperature. The luminescence signal was then measured using a plate reader (Tecan) and the luminescence signal for each treatment group was normalized to untreated wells. Percent cell viability is reported as the mean ± standard deviation of 4 technical replicates. A one-way ANOVA with post hoc Student’s t tests using the Holm-Šídák correction for multiple comparisons were used to compare cell viability across treatment groups.

### In vitro NF-κB activity assay

RAW-Blue cells (Invivogen, San Diego, CA) were cultured in DMEM (Gibco) supplemented with 10% FBS (Gibco) and 1% penicillin–streptomycin (Gibco). RAW-Blue cells were plated at a concentration of 80,000 cells per 100 µl of media, respectively, and left to adhere overnight followed by treatment with LNPs at a dose of 100 ng of mRNA. Twenty-four h after LNP treatment, NF-κB activity was evaluated by measuring secreted alkaline phosphatase levels in supernatant using a QUANTI-Blue Assay (InvivoGen) according to the manufacturer’s protocol. Normalized NF-κB activity was measured by normalizing to untreated cells. Results are reported as mean ± standard deviation from 4 biological replicates. A one-way ANOVA with post hoc Student’s t tests using the Holm- Šídák correction for multiple comparisons was used to compare normalized NF-κB activity across treatment groups.

### LNP uptake studies via fluorescence microscopy

HepG2, OVCAR8 and H1299 cells were plated at a concentration of 100,000 cells per 500 µl of media in 35 mm glass bottom dishes and left to adhere overnight. Cells were treated with DiI-labeled LNPs at a dose of 100 ng of mRNA. After 2 h, cells were stained with Hoechst 33342 (5 μg/mL, Thermo Fisher Scientific) for 10 min at 37 °C, washed with 1 × PBS, and stained with LysoTracker Deep Red (150 nM, Thermo Fisher Scientific) for 30 min at 37 °C. Finally, cells were incubated in phenol-red free DMEM or RPMI for imaging. Images were taken immediately using a confocal laser scanning microscope (Leica Stellaris 5).

Quantification of LNP colocalization and endosomal escape was performed by pixel-based analysis of fluorescence images. For each cell, a binary mask delineating the cell area was applied to all channels. Within this mask, LNP-positive pixels were identified in the DiI-LNP channel (G) using an intensity threshold that was kept constant for a given experiment. The mean G-channel intensity of all LNP-positive pixels inside the cell was recorded as MFI LNP (yellow), corresponding to the average DiI-LNP signal per LNP-positive pixel. Endolysosome-positive pixels were identified in the endolysosomal marker channel (R) using an analogous fixed threshold. The mean R-channel intensity of all endolysosome-positive pixels was recorded as MFI endolysosome (red). Pixels that were positive in both the LNP (G) and endolysosome (R) masks were classified as colocalized (LNP within endolysosomes). The mean G-channel intensity of these colocalized pixels was recorded as MFI coloc (orange), representing the LNP signal trapped inside endolysosomal compartments. To estimate endosomal escape, the contribution of LNP signal outside endolysosomes was calculated as the difference between total LNP MFI and colocalized LNP MFI. Endosomal escape for each cell was then expressed as the fraction of LNP MFI residing outside endolysosomes relative to the total (inside + outside) LNP MFI: *[MFI Pixel (LNP channel) – MFI Pixel (Colocalized Channel)]/[MFI Pixel (LNP channel)]*. Pearson’s correlation coefficient (r) was computed to quantify the degree of pixel-wise colocalization between DiI-labeled LNPs (yellow channel) and endolysosomal compartments (red channel) within individual cells. All analyses were performed using custom scripts in Fiji, following established fluorescence-colocalization quantification guidelines. Results are reported as mean ± standard deviation from 3 fields of view and 10 cells in each field of view. A one-way ANOVA with post hoc Student’s t tests using the Holm-Šídák correction for multiple comparisons was used to compare percent endosomal escape and Pearson coefficient across treatment groups.

### In vitro small molecule endocytosis inhibitor assays

HepG2, OVCAR8 and H1299 cells were plated at a concentration of 10,000, 20,000 or 10,000 cells per 100 µl of media, respectively, and left to adhere overnight. The endocytosis inhibitors amiloride (AMI, inhibitor of macropinocytosis, 200 mM, Sigma Aldrich), chlorpromazine (CPZ, inhibitor of clathrin-mediated endocytosis, 2000 µM, Sigma Aldrich), genistein (GEN, inhibitor of caveolae-mediated endocytosis, 20 mM, Sigma Aldrich), dynasore (DYNA, inhibitor of dynamin-dependent endocytosis, 10 mM, Sigma Aldrich), methyl-beta-cyclodextrin (MBCD, inhibitor of lipid-raft mediated endocytosis, 500 mM, Sigma Aldrich) were dissolved in DMSO at their respective concentrations. After overnight adherence, 1µL of each endocytosis inhibitor (Amiloride (AMI), Chlorpromazine (CPZ), Genistein (GEN), Dynasore (DYNA), Methyl-beta-cyclodextrin (MBCD)) was added to the appropriate wells and incubated for 30 min. After incubation with each inhibitor, cells were resuspended in fresh DMEM or RPMI medium and dosed with 10 ng of luciferase mRNA LNPs for HepG2 cells, 25 ng of luciferase mRNA LNPs for OVCAR8 cells and 20 ng of luciferase mRNA LNPs for H1299 cells. Twenty-four  h after treatment with LNPs, the luminescence signal was measured as previously described. Relative luminescence signal was calculated by first subtracting the background readings from untreated cells and then dividing by the average luminescence signal of cells treated with LNPs without inhibitor for each LNP treatment group. Relative luciferase expression is reported as the mean ± standard deviation of 3 biological replicates (averaged from 3 technical replicates). A two-way ANOVA with post hoc Student’s t tests using the Holm-Šídák correction for multiple comparisons was used to compare the results across treatment groups and inhibitors and nested t-tests were used to compare luciferase expression for each LNP and inhibitor treatment groups to cells treated with no inhibitor.

## Supplementary Information

Supplementary Material 1.

## Data Availability

The data that support the findings of this study are available from the corresponding author upon reasonable request.
