# Supplementary material for: Examining the effect of lipid nanoparticle elasticity on endocytosis and mRNA delivery to cancer cells
Source: Med X. 2026 May 1;4(1):11. doi: 10.1007/s44258-026-00082-w (PMC13132934; doi:10.1007/s44258-026-00082-w)
Supplement: Supplementary file 1 — Supplementary Material 1. [file 44258_2026_82_MOESM1_ESM.docx]

# Examining the effect of lipid nanoparticle elasticity on endocytosis and mRNA delivery to cancer cells

Cecilia F. Shuler^1†^, Hannah C. Safford^2†^, Ajay S. Thatte^2^, Melgious Ang^2,3^, Michael J. Mitchell^3,4,5,6,7,8^*

^1^Department of Biophysics, University of Pennsylvania, Philadelphia, Pennsylvania, 19104, USA

^2^Department of Bioengineering, University of Pennsylvania, Philadelphia, Pennsylvania, 19104, USA

^3^Bioprocessing Technology Institute (BTI), Agency for Science, Technology and Research (A*STAR), Republic of Singapore, 138669, Singapore.

^4^Penn Institute for RNA Innovation, Perelman School of Medicine, University of Pennsylvania, Philadelphia, Pennsylvania, 19104, USA

^5^Abramson Cancer Center, Perelman School of Medicine, University of Pennsylvania, Philadelphia, Pennsylvania, 19104, USA

^6^Institute for Immunology, Perelman School of Medicine, University of Pennsylvania, Philadelphia, Pennsylvania, 19104, USA

^7^Cardiovascular Institute, Perelman School of Medicine, University of Pennsylvania, Philadelphia, Pennsylvania, 19014, USA

^8^Institute for Regenerative Medicine, Perelman School of Medicine, University of Pennsylvania, Philadelphia, Pennsylvania, 19104, USA

†These authors contributed equally to this work.

**Corresponding Author**

[*mjmitch@seas.upenn.edu](mailto:*mjmitch@seas.upenn.edu)

**SI Figures**


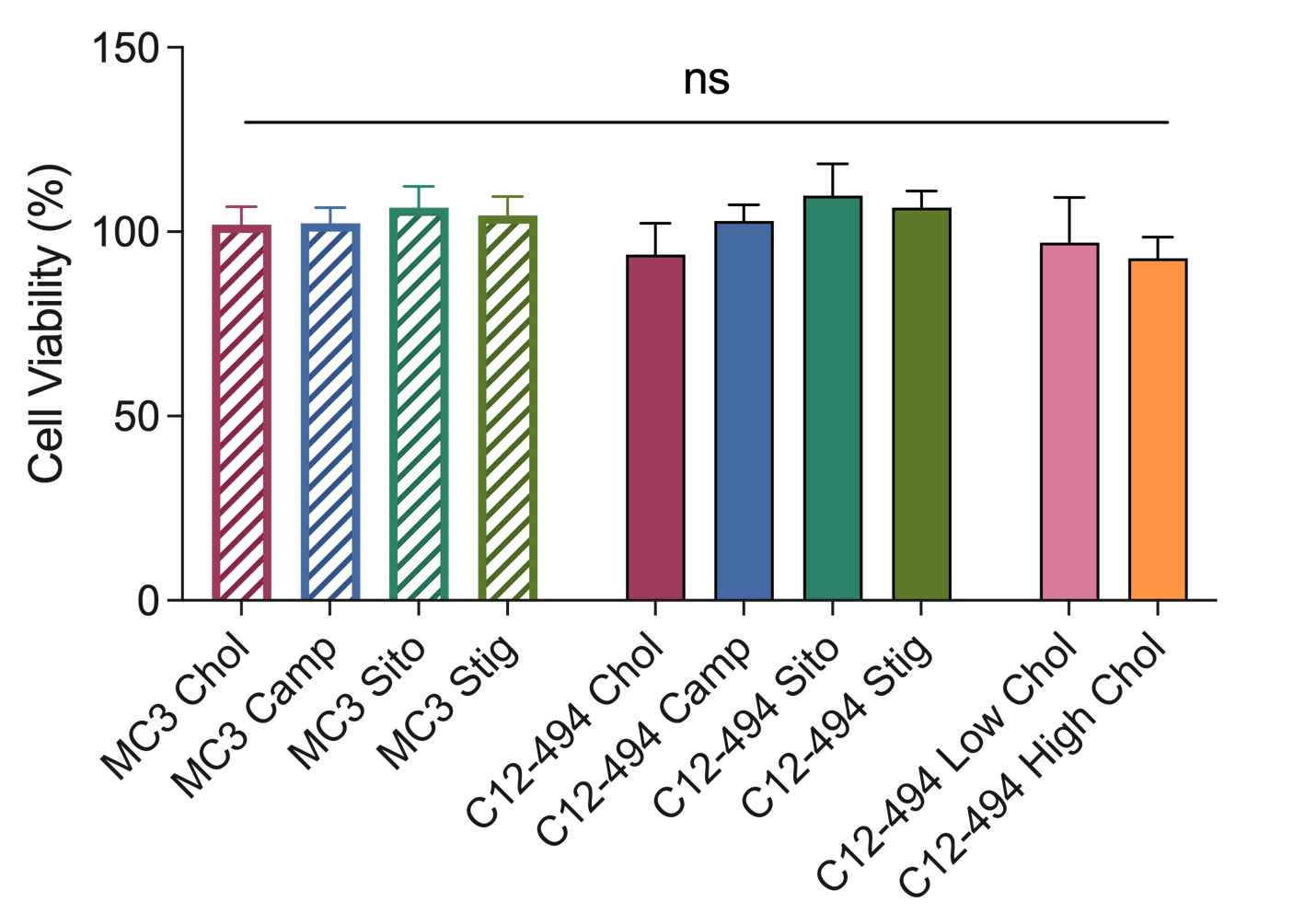


**Fig. S1**. Cell viability of HepG2 cells 24 h after treatment with MC3 Chol, Camp, Sito and Stig LNPs, C12-494 Chol, Camp, Sito and Stig LNPs, and C12-494 Low Chol and High Chol LNPs at a dose of 10 ng of mRNA per 10,000 cells. Cell viability was measured by normalizing to untreated cells. Results are reported as mean ± standard deviation from n = 4 biological replicates. A one-way ANOVA with post hoc Student’s t tests using the Holm- Šídák correction for multiple comparisons was used to compare cell viability across treatment groups, ns = not significant.


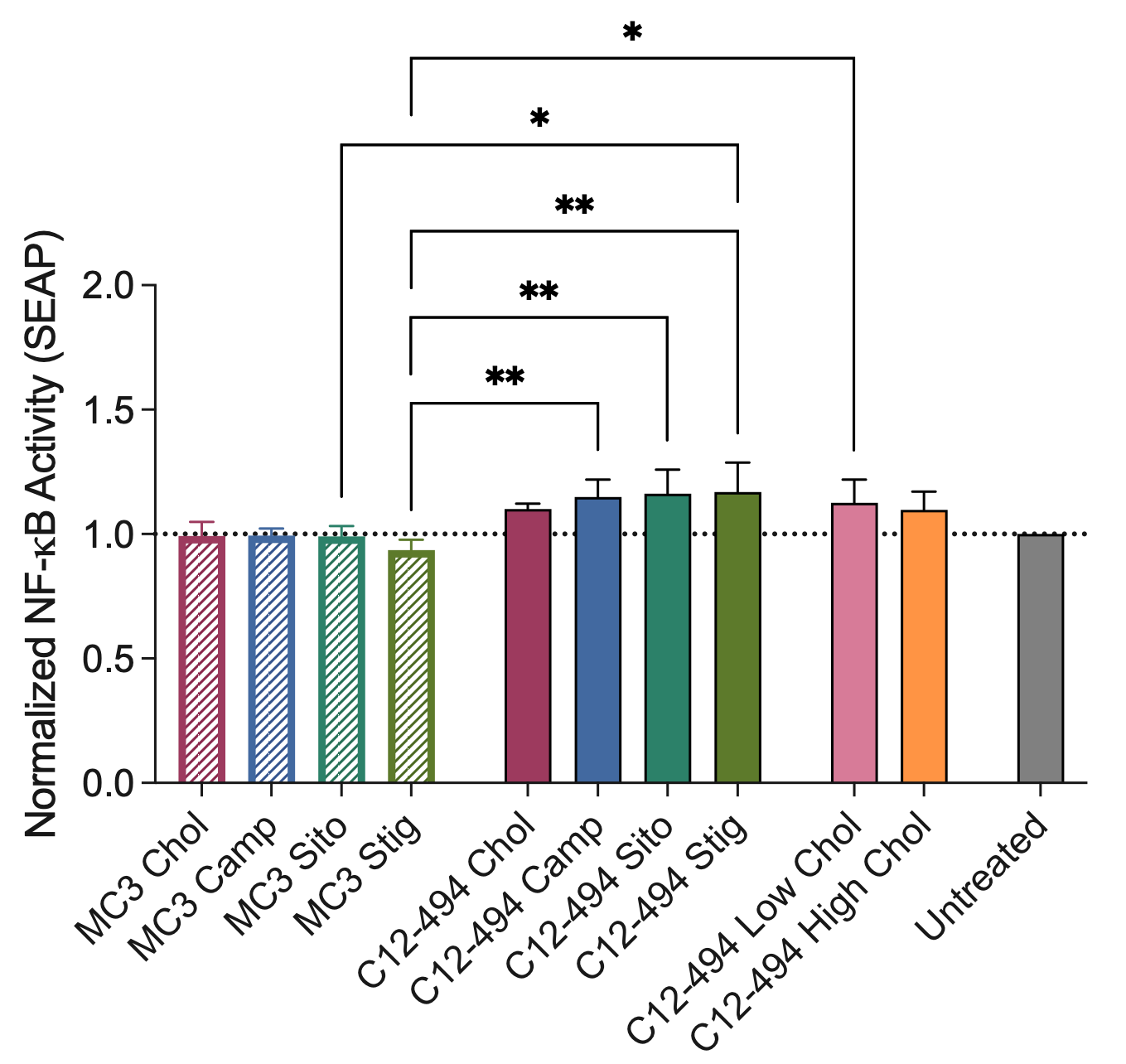


**Fig. S2.** Quantification of NF-κB activity in RAW-Blue cells 24 h after treatment with MC3 Chol, Camp, Sito and Stig LNPs, C12-494 Chol, Camp, Sito and Stig LNPs, and C12-494 Low Chol and High Chol LNPs at a dose of 100 ng of mRNA per 80,000 cells. Normalized NF-κB activity was measured by normalizing to untreated cells. Results are reported as mean ± standard deviation from n = 4 biological replicates. A one-way ANOVA with post hoc Student’s t tests using the Holm- Šídák correction for multiple comparisons was used to compare normalized NF-κB activity across treatment groups, *p ≤ 0.05, **p ≤ 0.01.


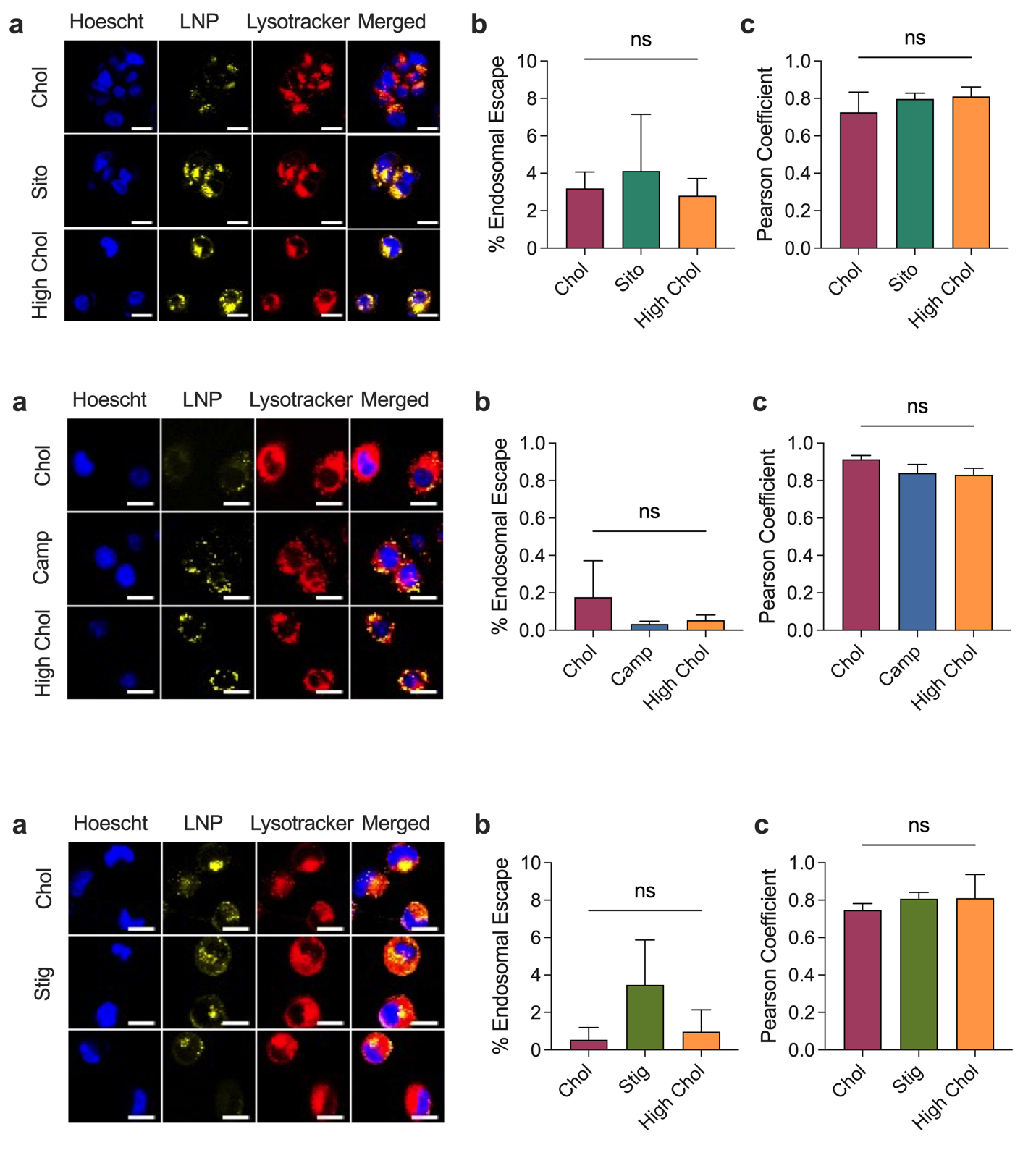


**Fig. S3. (a)** Representative confocal microscopy images of DiI-labeled LNP uptake in HepG2 cells 2 hours after treatment with C12-494 Chol, Sito and High Chol LNPs. Scale bar: 10 μm. **(b)** Quantification of percent endosomal escape, as quantified by percent delocalized signal between DiI-labeled LNPs and lysotracker-labeled endosomes, and **(c)** corresponding Pearson correlation coefficient of the LNP formulations. Results are reported as mean ± standard deviation from n = 3 fields of view and n = 10 cells in each field of view. A one-way ANOVA with post hoc Student’s t tests using the Holm-Šídák correction for multiple comparisons was used to compare percent endosomal escape and Pearson coefficient across treatment groups, ns = not significant.


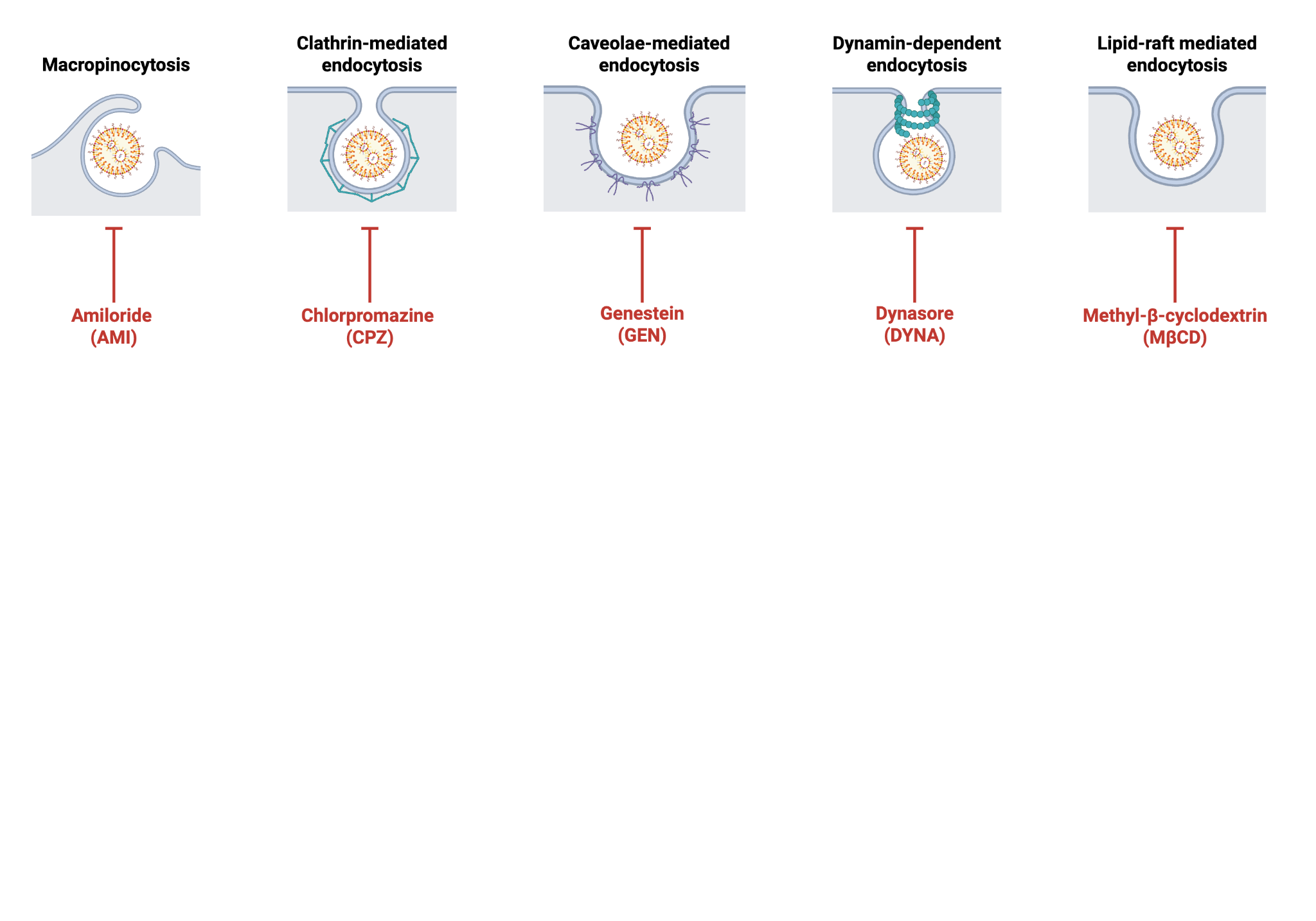


**Fig. S4**. Schematic of different endocytosis pathways with their respective inhibitors.


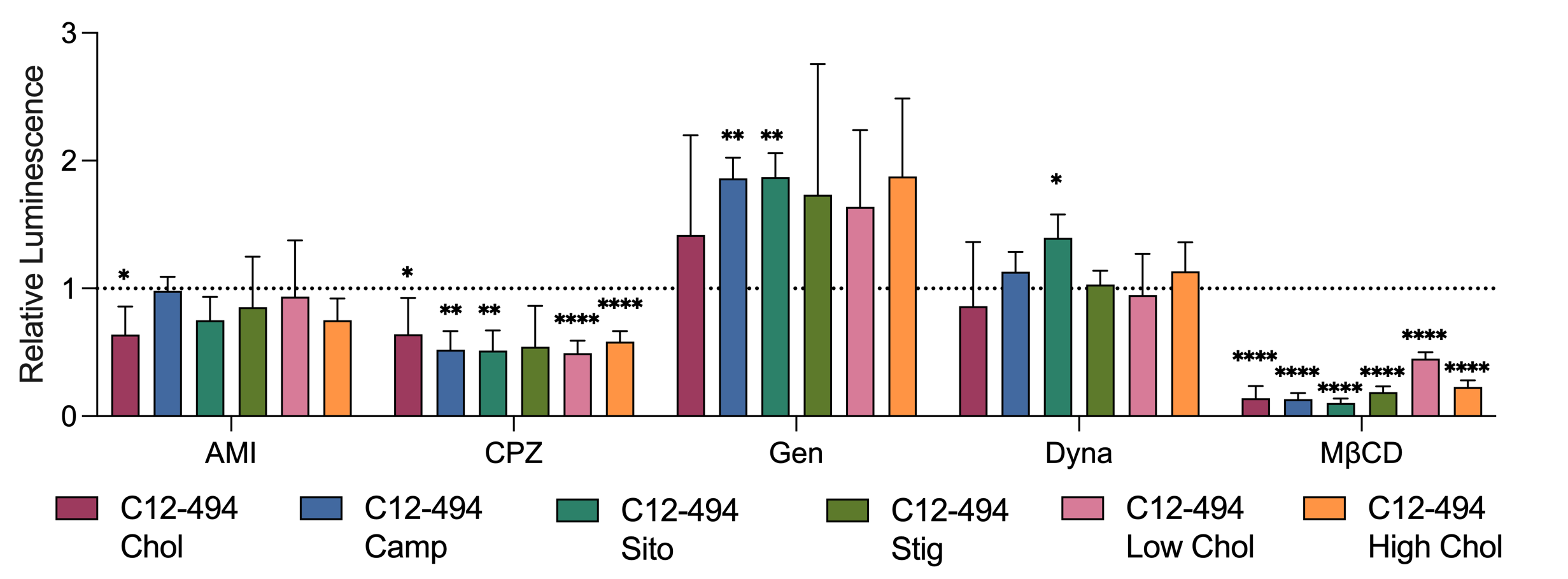


**Fig. S5**. Relative luciferase expression in HepG2 cells 24 hours after treatment with C12-494 LNPs incorporating each cholesterol analog or C12-494 low and high cholesterol LNPs at a dose of 10 ng of mRNA per 10,000 cells in the presence of different endocytosis inhibitors (Amiloride (AMI) is an inhibitor of macropinocytosis; chlorpromazine (CPZ) is an inhibitor of clathrin-mediated endocytosis; genistein (GEN) is an inhibitor of caveolae-mediated endocytosis; dynasore (DYNA) is an inhibitor of dynamin-dependent endocytosis; methyl-β-cyclodextrin (MβCD) is an inhibitor of lipid-raft mediated endocytosis). Relative luminescence signal was quantified by normalizing to cells treated with LNPs in the absence of endocytosis inhibitors. Results are reported as mean ± standard deviation from n = 3 biological replicates. Nested t-tests were used to compare luciferase expression for each LNP and inhibitor treatment groups to cells treated with no inhibitor (dashed line), *p ≤ 0.05, **p ≤ 0.01, ***p ≤ 0.001, ****p ≤ 0.0001.


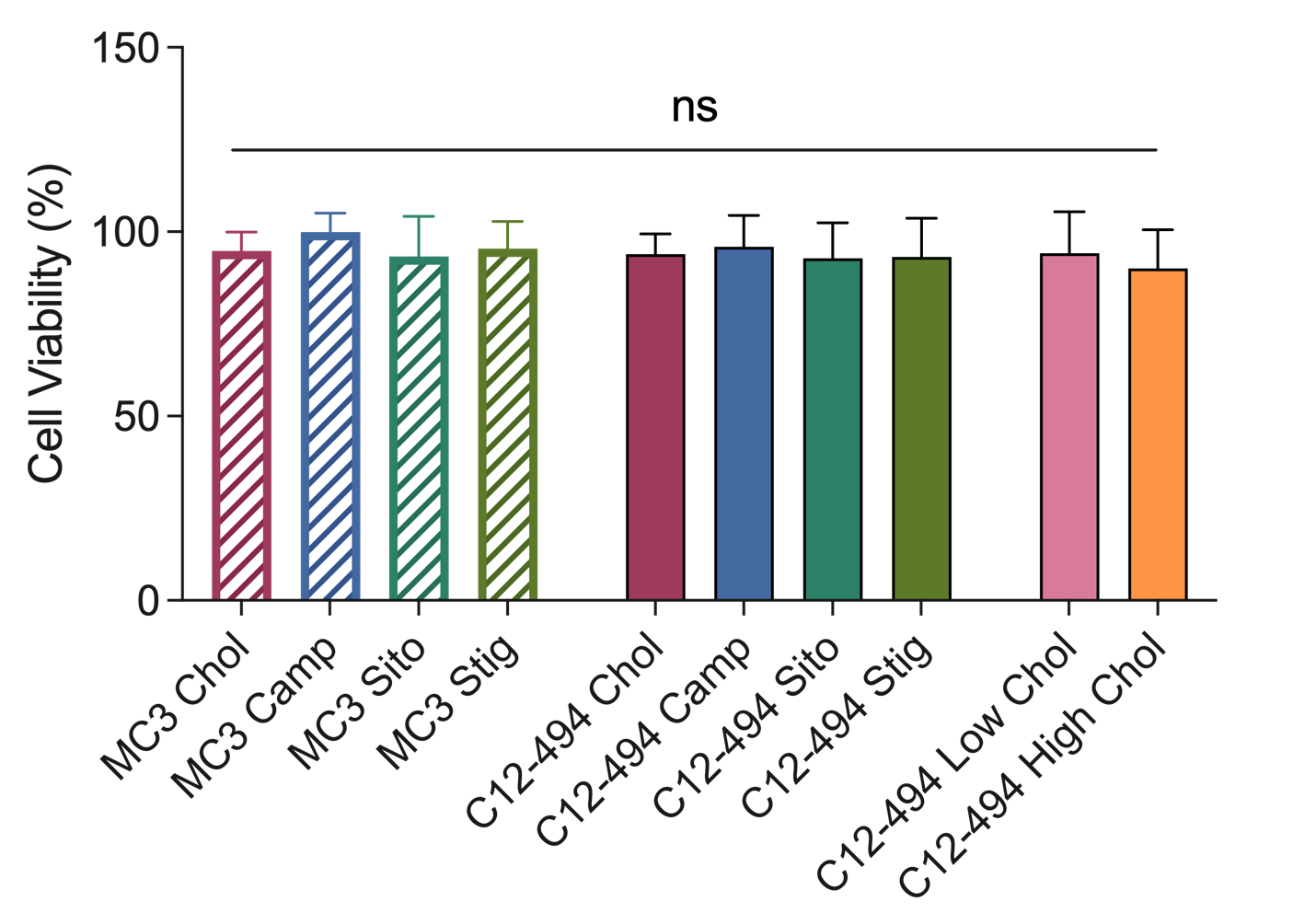


**Fig. S6**. Cell viability of OVCAR8 cells 24 h after treatment with MC3 Chol, Camp, Sito and Stig LNPs, C12-494 Chol, Camp, Sito and Stig LNPs, and C12-494 Low Chol and High Chol LNPs at a dose of 25 ng of mRNA per 20,000 cells. Cell viability was measured by normalizing to untreated cells. Results are reported as mean ± standard deviation from n = 4 biological replicates. A one-way ANOVA with post hoc Student’s t tests using the Holm- Šídák correction for multiple comparisons was used to compare cell viability across treatment groups, ns = not significant.


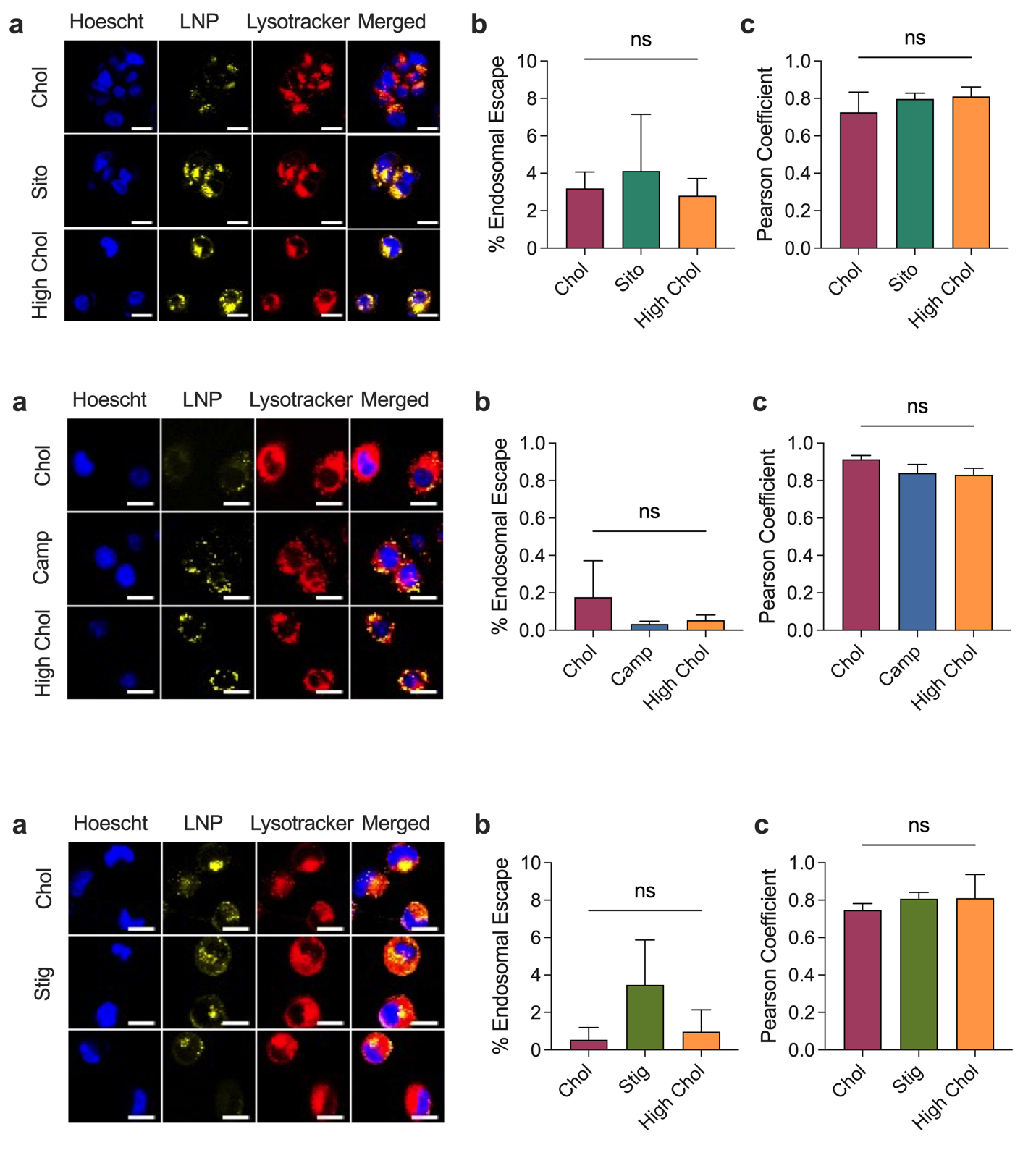


**Fig. S7. (a)** Representative confocal microscopy images of DiI-labeled LNP uptake in OVCAR8 cells 2 hours after treatment with C12-494 Chol, Camp and High Chol LNPs. Scale bar: 10 μm. **(b)** Quantification of percent endosomal escape, as quantified by percent delocalized signal between DiI-labeled LNPs and lysotracker-labeled endosomes, and **(c)** corresponding Pearson correlation coefficient of the LNP formulations. Results are reported as mean ± standard deviation from n = 3 fields of view and n = 10 cells in each field of view. A one-way ANOVA with post hoc Student’s t tests using the Holm-Šídák correction for multiple comparisons was used to compare percent endosomal escape and Pearson coefficient across treatment groups, ns = not significant.


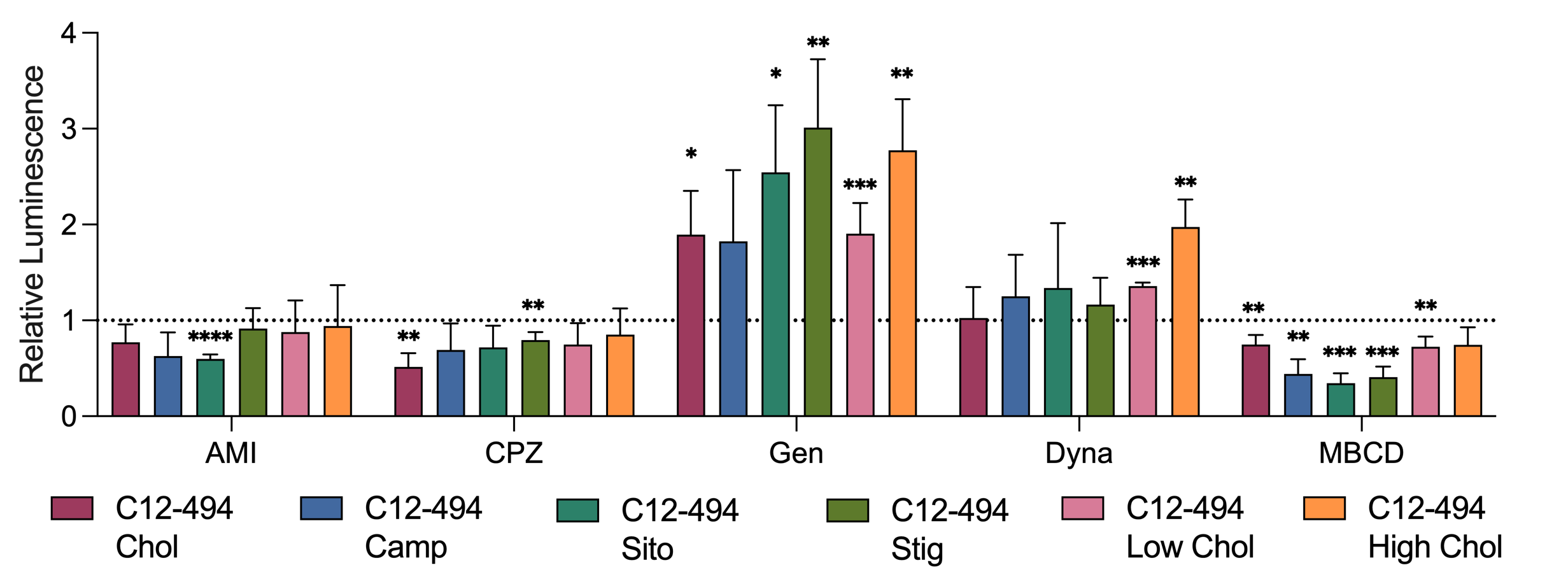


**Fig. S8**. Relative luciferase expression in OVCAR8 cells 24 hours after treatment with C12-494 LNPs incorporating each cholesterol analog or C12-494 low and high cholesterol LNPs at a dose of 25 ng of mRNA per 20,000 cells in the presence of different endocytosis inhibitors (Amiloride (AMI) is an inhibitor of macropinocytosis; chlorpromazine (CPZ) is an inhibitor of clathrin-mediated endocytosis; genistein (GEN) is an inhibitor of caveolae-mediated endocytosis; dynasore (DYNA) is an inhibitor of dynamin-dependent endocytosis; methyl-β-cyclodextrin (MβCD) is an inhibitor of lipid-raft mediated endocytosis). Relative luminescence signal was quantified by normalizing to cells treated with LNPs in the absence of endocytosis inhibitors. Results are reported as mean ± standard deviation from n = 3 biological replicates. Nested t-tests were used to compare luciferase expression for each LNP and inhibitor treatment groups to cells treated with no inhibitor (dashed line), *p ≤ 0.05, **p ≤ 0.01, ***p ≤ 0.001, ****p ≤ 0.0001.


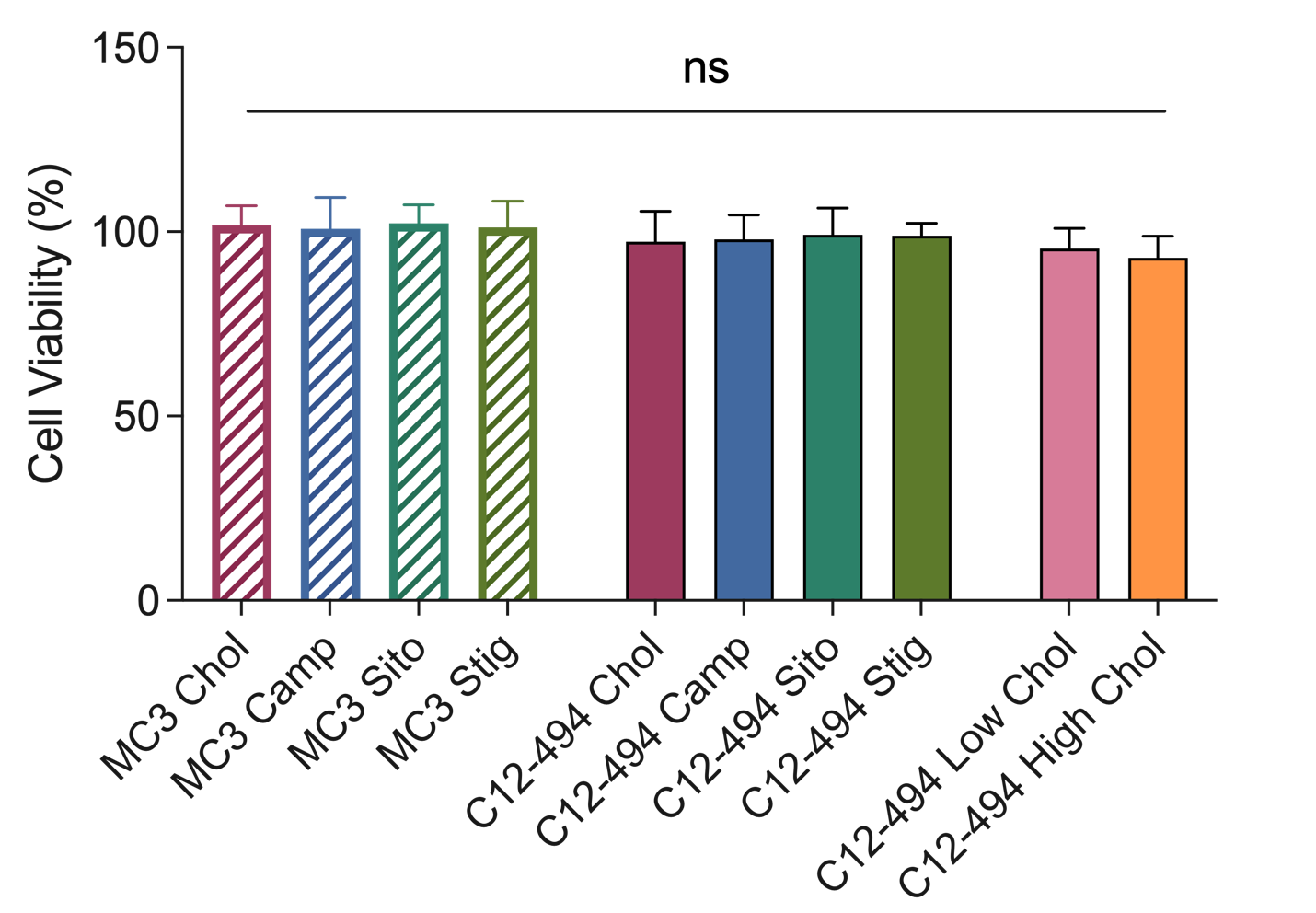


**Fig. S9**. Cell viability of H1299 cells 24 h after treatment with MC3 Chol, Camp, Sito and Stig LNPs, C12-494 Chol, Camp, Sito and Stig LNPs, and C12-494 Low Chol and High Chol LNPs at a dose of 20 ng of mRNA per 20,000 cells. Cell viability was measured by normalizing to untreated cells. Results are reported as mean ± standard deviation from n = 4 biological replicates. A one-way ANOVA with post hoc Student’s t tests using the Holm- Šídák correction for multiple comparisons was used to compare cell viability across treatment groups, ns = not significant.


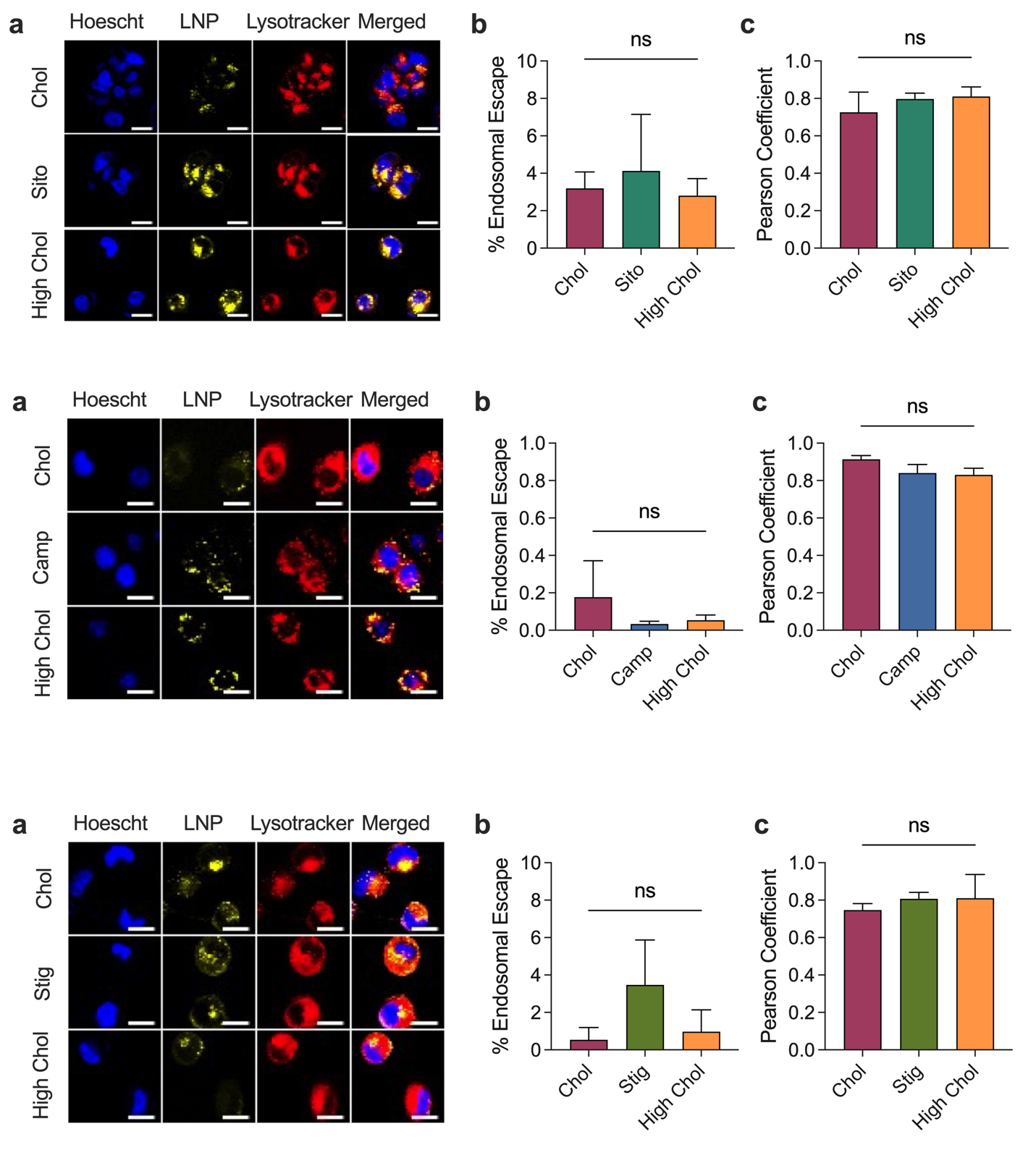


**Fig. S10. (a)** Representative confocal microscopy images of DiI-labeled LNP uptake in H1299 cells 2 hours after treatment with C12-494 Chol, Stig and High Chol LNPs. Scale bar: 10 μm. **(f)** Quantification of percent endosomal escape, as quantified by percent delocalized signal between DiI-labeled LNPs and lysotracker-labeled endosomes, and **(g)** corresponding Pearson correlation coefficient of the LNP formulations. Results are reported as mean ± standard deviation from n = 3 fields of view and n = 10 cells in each field of view. A one-way ANOVA with post hoc Student’s t tests using the Holm-Šídák correction for multiple comparisons was used to compare percent endosomal escape and Pearson coefficient across treatment groups, ns = not significant.


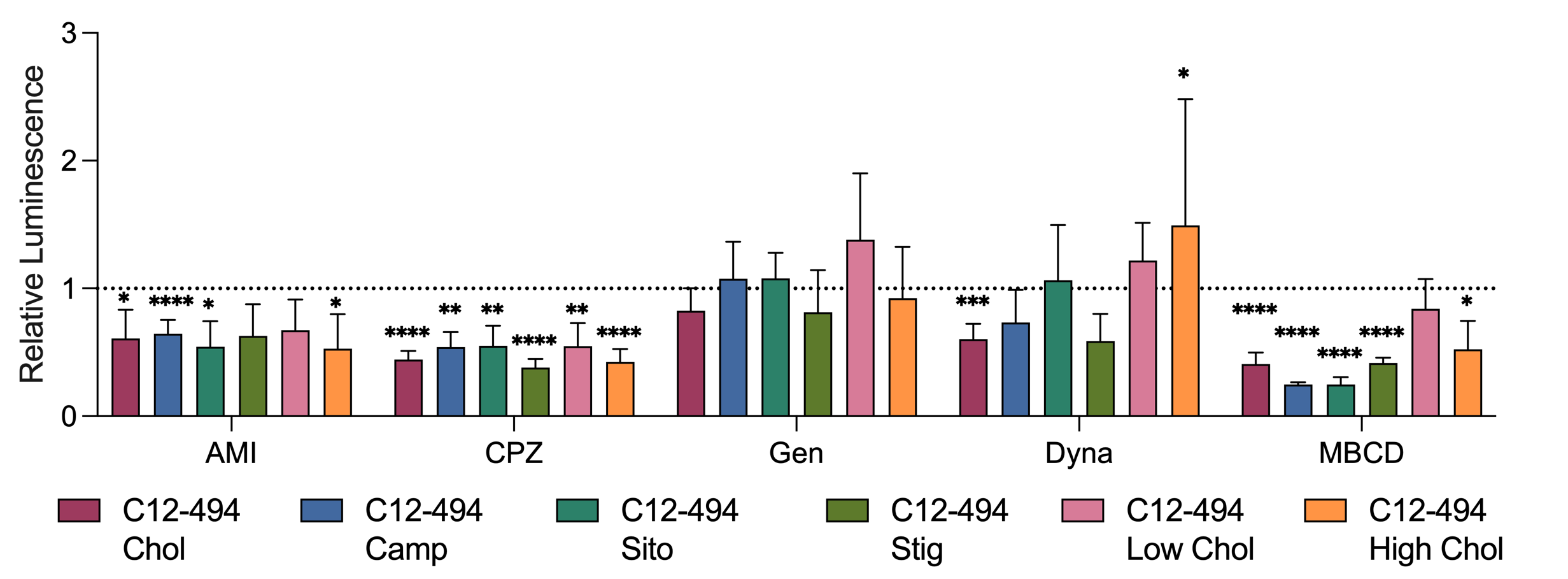


**Fig. S11**. Relative luciferase expression in H1299 cells 24 hours after treatment with C12-494 LNPs incorporating each cholesterol analog or C12-494 low and high cholesterol LNPs at a dose of 20 ng of mRNA per 20,000 cells in the presence of different endocytosis inhibitors (Amiloride (AMI) is an inhibitor of macropinocytosis; chlorpromazine (CPZ) is an inhibitor of clathrin-mediated endocytosis; genistein (GEN) is an inhibitor of caveolae-mediated endocytosis; dynasore (DYNA) is an inhibitor of dynamin-dependent endocytosis; methyl-β-cyclodextrin (MβCD) is an inhibitor of lipid-raft mediated endocytosis). Relative luminescence signal was quantified by normalizing to cells treated with LNPs in the absence of endocytosis inhibitors. Results are reported as mean ± standard deviation from n = 3 biological replicates. Nested t-tests were used to compare luciferase expression for each LNP and inhibitor treatment groups to cells treated with no inhibitor (dashed line), *p ≤ 0.05, **p ≤ 0.01, ***p ≤ 0.001, ****p ≤ 0.0001.

# **SI Tables:**

**Table S1:** Formulation details for LNPs used for *in vitro* screening. The molar ratio is shown as ionizable lipid:phospholipid:sterol:lipid-PEG.

| Formulation | Ionizable Lipid (IL) | Phospholipid (P) | Sterol (S) | Lipid-PEG (PEG) | Molar ratio (IL:P:S:PEG) |
| --- | --- | --- | --- | --- | --- |
| MC3 Chol | MC3 | DSPC | Cholesterol | DMG-PEG_2k_ | 50:10:38.5:1.5 |
| MC3 Camp | MC3 | DSPC | Campesterol | DMG-PEG_2k_ | 50:10:38.5:1.5 |
| MC3 Sito | MC3 | DSPC | β-sitosterol | DMG-PEG_2k_ | 50:10:38.5:1.5 |
| MC3 Stig | MC3 | DSPC | Stigmastanol | DMG-PEG_2k_ | 50:10:38.5:1.5 |
| C12-494 Chol | C12-494 | DOPE | Cholesterol | C14-PEG_2k_ | 35:16:46.5:2.5 |
| C12-494 Camp | C12-494 | DOPE | Campesterol | C14-PEG_2k_ | 35:16:46.5:2.5 |
| C12-494 Sito | C12-494 | DOPE | β-sitosterol | C14-PEG_2k_ | 35:16:46.5:2.5 |
| C12-494 Stig | C12-494 | ​​DOPE | Stigmastanol | C14-PEG_2k_ | 35:16:46.5:2.5 |
| C12-494 Low Chol | C12-494 | DOPE | Cholesterol | C14-PEG_2k_ | 35:16:31.5:2.5 |
| C12-494 High Chol | C12-494 | DOPE | Cholesterol | C14-PEG_2k_ | 35:16:61.5:2.5 |

**Table S2:** Size, PDI, encapsulation efficiency, and zeta potential for LNPs used for *in vitro* screening.

| **Formulation** | **Z-average Size (nm)** | **Polydispersity Index** | **Encapsulation Efficiency (%)** | **Zeta Potential (mV)** |
| --- | --- | --- | --- | --- |
| MC3 Cholesterol | 73.9±1.1 | 0.08±0.01 | 96.4±0.7 | -13.56±1.23 |
| MC3 Campesterol | 72.1±0.8 | 0.14±0.03 | 95.9±1.2 | -21.09±0.66 |
| MC3 β-sitosterol | 84.8±0.9 | 0.07±0.02 | 95.3±2.6 | -16.63±0.29 |
| MC3 Stigmastanol | 83.4±0.5 | 0.07±0.01 | 92.8±6.1 | -15.35±0.47 |
| C12-494 Cholesterol | 78.7±0.8 | 0.23±0.04 | 93.7±2.4 | -6.20±2.53 |
| C12-494 Campesterol | 76.4±1.1 | 0.26±0.01 | 91.9±4.1 | -2.92±0.92 |
| C12-494 β-sitosterol | 84.7±0.2 | 0.31±0.01 | 92.7±7.3 | -8.24±4.92 |
| C12-494 Stigmastanol | 70.6±0.2 | 0.23±0.02 | 92.4±4.5 | -4.80±1.08 |
| C12-494 Low Cholesterol | 78.0±1.4 | 0.13±0.02 | 94.8±2.5 | -2.70±0.34 |
| C12-494 High Cholesterol | 87.7±1.2 | 0.14±0.02 | 93.2±7.1 | -5.22±0.33 |
